# Investigation of a Large Collection of *Pseudomonas aeruginosa* Bacteriophages Collected from a Single Environmental Source in Abidjan, Côte d’Ivoire

**DOI:** 10.1371/journal.pone.0130548

**Published:** 2015-06-26

**Authors:** Christiane Essoh, Libera Latino, Cédric Midoux, Yann Blouin, Guillaume Loukou, Simon-Pierre A. Nguetta, Serge Lathro, Arsher Cablanmian, Athanase K. Kouassi, Gilles Vergnaud, Christine Pourcel

**Affiliations:** 1 Institute for Integrative Biology of the Cell, CEA, CNRS, Univ Paris-Sud, Université Paris-Saclay, Orsay, France; 2 Laboratoire National de Santé Publique, Abidjan, Côte d’Ivoire; 3 Laboratoire de Bactériologie-Virologie, département de Sciences pharmaceutiques et Biologiques, Univ Félix Houphouët-Boigny, Abidjan, Côte d’Ivoire; 4 Laboratoire de Génétique, Département des Biosciences, Univ Félix Houphouet-Boigny, Abidjan, Côte d’Ivoire; 5 ENSTA ParisTech, Université Paris-Saclay, Palaiseau, France; Institute of Immunology and Experimental Therapy, Polish Academy of Sciences, POLAND

## Abstract

Twenty two distinct bacteriophages were isolated from sewage water from five locations in the city of Abidjan, Côte d'Ivoire over a two-year period, using a collection of *Pseudomonas aeruginosa* strains with diverse genotypes. The phages were characterized by their virulence spectrum on a panel of selected *P*. *aeruginosa* strains from cystic fibrosis patients and by whole genome sequencing. Twelve virions representing the observed diversity were visualised by electron microscopy. The combined observations showed that 17 phages, distributed into seven genera, were virulent, and that five phages were related to temperate phages belonging to three genera. Some showed similarity with known phages only at the protein level. The vast majority of the genetic variations among virulent phages from the same genus resulted from seemingly non-random horizontal transfer events, inside a population of *P*. *aeruginosa* phages with limited diversity. This suggests the existence of a single environmental reservoir or ecotype in which continuous selection is taking place. In contrast, mostly point mutations were observed among phages potentially capable of lysogenisation. This is the first study of *P*. *aeruginosa* phage diversity in an African city and it shows that a large variety of phage species can be recovered in a limited geographical site at least when different bacterial strains are used. The relative temporal and spatial stability of the Abidjan phage population might reflect equilibrium in the microbial community from which they are released.

## Introduction

Bacteriophages, viruses that infect bacteria, are the most numerous of all viruses in the biosphere and are estimated to be globally more numerous than bacteria (reviewed in [1,2,3]). This abundance plays important roles in the evolution of bacterial communities and may influence global biogeochemical cycles [4]. A limited number of studies have investigated phage distribution in natural environments, in contrast to the vast knowledge on bacterial ecology [5]. Man-made environments such as waste water systems are rich in microbial communities due to diversified sources of microbes, presence of elevated level of nutrients and the multiple surfaces on which biofilms can form [6]. Different studies have revealed that such ecosystems can be reservoir for human pathogens and associated bacteriophages [7,8].

Evolution of phages, driven by resistance of bacterial hosts, can affect multiple genes, and this is reflected by the large genomic diversity inside phage species [9,10]. Comparison of genomes reveals important levels of mosaicism resulting from recombination, as well as acquisition or loss of genetic material and point mutations [11]. It has been speculated that the recombination functions encoded by many phages are, in addition to their role for phage replication, responsible for the creation and the maintenance of mosaicism. Their activity would re-create different modules with a comparable selective fitness [12]. Viruses constitute a huge reservoir of genetic diversity that is frequently revealed by the discovery of novel genes especially in newly sequenced phage genomes [13]. Metagenomic data from aquatic and human environments show that most viral diversity remains uncharacterized [14,15].

Ubiquitous in the environment, *P*. *aeruginosa* is one of the major life-threatening opportunistic bacteria responsible for nosocomial infections in immunocompromised people, and for persistent respiratory infections in cystic fribrosis (CF) patients [16,17]. Its ability to adapt to different niches and to develop resistance to classical antibiotic-based therapy has inspired a renewed interest in its bacteriophages. Phage cocktails are used to treat *P*. *aeruginosa* infections in different countries but clinical assays are needed to evaluate their efficiency and safety. The composition of therapeutic cocktails such as the widely used Pyo-Phage [18] remains empirical, and there is no clear and published rules on how many phages should be included, and what would be the criteria for selecting them.

Considering the large genetic diversity of the *P*. *aeruginosa* species, it is important for future therapeutic usage to evaluate the virulence spectrum of lytic phages [19,20]. Numerous lytic and temperate *P*. *aeruginosa* phages have been isolated, mostly from man-made environments of European and Asian countries, and new ones are continuously being described [21,22]. Ceyssens et al. analyzing morphologic features and genome organization of a large collection of phages, estimated that most evolved within 12 genera gathering 21 species [10]. Two additional genera of lytic phages were subsequently described. The PAK-P1-like phages include JG004, vB_PaeM_C2-10_Ab1 (alias Ab01) and PaP1 [23,24,25,26], whereas KPP10 and PAK-P3 form a second genus [27,28]. More recently six new species belonging to the *Siphoviridae* family were reported [7], as well as a giant phage [29]. The large majority of siphoviruses of *P*. *aeruginosa* has been shown to be temperate phages distributed into three different genera, implying the possibility to undergo a lytic or a lysogenic interaction with their host [30]. Such phages often show a very narrow host range. The recently isolated lytic siphovirus PA1ɸ, a D3112-like phage lacking lysogeny gene modules, displayed a broad bacteria spectrum [31]. Among podoviruses, the Abidjan phage Ab31, a chimera between a temperate and a lytic phage, was shown to be temperate [26].To understand the mechanisms that shape bacteriophages populations and allow the emergence of new species by mutations and genome exchanges, it is necessary to perform studies resampling the same environment. We previously described a large panel of clinical *P*. *aeruginosa* isolates that showed a wide variety of susceptibility to lytic and temperate phages [26]. Here we used a selection of these strains to isolate and characterize 22 novel *P*. *aeruginosa* phages from sewage water in Abidjan (Ivory Coast) in 2010 and 2011. Comparison of their genomes to those of published phages reveals a large diversity and points to a continuous evolution in a limited time and space scale.

## Materials and Methods

### Ethics statement

The present project is in compliance with the Helsinki Declaration (Ethical Principles for Medical Research Involving Human Subjects). Bacterial strains were collected as part of the patients' usual care, without any additional sampling, as previously reported [32,33,34]. The ethic committee “Comité Consultatif pour la Protection des Personnes dans la Recherche Biomédicale (CCPPRB) Ile-de-France”, who was consulted, specifically approved this study, and declared that patient informed consent was not needed.

### Strains and media

The *P*. *aeruginosa* strains used for enrichment and amplification of phages were from CF patients (Table 1) [32,33,34]. The reference strains PAO1 and PA14 were purchased from the Institut Pasteur Collection (CIP, Paris, France). C50, a strain from clone C [35] was a gift from Ute Römling. Twelve *P*. *aeruginosa* strains were used for phage isolation, of which six were resistant to Pyo-Phage (a therapeutic cocktail of phages prepared in Tbilisi, Georgia [18]), and to additional phages from Russia and USA ([26] and unpublished results). Five bacterial strains were used for subsequent amplification of the purified phages. Phages p1-14_pyo_, p8_13_pyo_, p1-15_pyo_ and p2-10_pyo_ were previously isolated from Pyo-Phage and were used in the present study as controls [26]. Luria broth (LB) medium supplemented with 2 mM CaCl_2_ was used for bacterial growth and phage titration. Saline magnesium (SM) buffer (50 mM Tris-HCl pH7.5, 100 mM NaCl, 8.1 mM MgSO_4_, 0.01% gelatin) was used to preserve purified phages at 4°C.

**Table 1:** List of bacteriophages isolated in Abidjan, Côte d’Ivoire.

Phage ID	Alias	Site^a^	Date	Enrichment strain ^b^
vB_PaeM_C2-10_Ab1	Ab01^c^	CV	oct-10	C2-10 (+)
vB_PaeM_C2-10_Ab02	Ab02	Ind	nov-11	C7-6 (-)
vB_PaeM_PAO1_Ab03	Ab03	CoH	nov-11	PAO1 (+)
vB_PaeM_PAO1_Ab04	Ab04	Ind	nov-11	C5-2 (-)
vB_PaeP_PAO1_Ab05	Ab05	CoH	nov-11	PAO1 (+)
vB_PaeM_PAO1_Ab06	Ab06	TrH	nov-11	PAO1 (+)
vB_PaeM_C2-10_Ab07	Ab07	CV	oct-10	C9-11 (+)
vB_PaeM_C2-10_Ab08	Ab08	Ind	oct-10	C9-11 (+)
vB_PaeP_C2-10_Ab09	Ab09	Ind	oct-10	SCH (+)
vB_PaeM_C2-10_Ab10	Ab10	CV	oct-10	SCH (+)
vB_PaeM_PAO1_Ab11	Ab11	CV	oct-10	PAO1 (+)
vB_PaeP_C5-2_Ab12^d^	Ab12	Ind	nov-11	C5-2 (-)
vB_PaeM_C2-10_Ab13	Ab13	Ba	oct-10	C2-10 (+)
vB_PaeM_C2-10_Ab14	Ab14	Ba	oct-10	C2-10 (+)
vB_PaeM_C2-10_Ab15	Ab15	CoH	oct-10	C9-11 (+)
vB_PaeM_C2-10_Ab16	Ab16	CV	oct-10	C9-11 (+)
vB_PaeM_PAO1_Ab17	Ab17	Ind	nov-11	PAO1 (+)
vB_PaeS_PAO1_Ab18	Ab18	CV	oct-10	PAO1 (+)
vB_PaeS_PAO1_Ab19	Ab19	CV	oct-10	C50 (-)
vB_PaeS_PAO1_Ab20	Ab20	Ind	oct-10	PAO1 (+)
vB_PaeS_PAO1_Ab21	Ab21	Ind	oct-10	PAO1 (+)
vB_PaeP_C2-10_Ab22	Ab22	Ind	nov-11	C8-20 (-)
vB_PaeM_C2-10_Ab23	Ab23	CV	oct-10	C8-20 (-)
vB_PaeM_C2-10_Ab24	Ab24	Ba	oct-10	C8-14 (-)
vB_PaeM_C2-10_Ab25	Ab25	CV	oct-10	SCH (+)
vB_PaeS_SCH_Ab26	Ab26	CV	oct-10	SCH (+)
vB_PaeM_PAO1_Ab27	Ab27	CoH	oct-10	C8-14 (-)
vB_PaeM_PAO1_Ab28	Ab28	Ind	oct-10	C8-14 (-)
vB_PaeM_PAO1_Ab29	Ab29	Ind	oct-10	C3-16 (-)
vB_PaeS_PAO1_Ab30	Ab30	CV	oct-10	C3-16 (-)
vB_PaeP_Tr60_Ab31^e^	Ab31	TrH	nov-11	PA14 (+)

^a^ CV, Carrefour de la Vie: Ind, indénié: CoH, Cocody Hospital; TrH, Treichville hospital; Ba, La Baie

^b^ in parentheses is indicated the Pyo-Phage-susceptibility

^c^ published in [26]

^d^ Ab12 was subsequently lost

^e^ published in [36]

### Phage isolation, characterization and genome sequencing

Samples were collected in five public sites: four in the Cocody area (La Baie, latitude 5.333704 longitude: -4.015846, Carrefour de la Vie, lat. 5.348505 long. -4.002178, Carrefour de l’Indénié, lat. 5.341143 long -4.017788, Cocody hospital, lat. 5.344389 long. -3.994072) and one in the Treichville area (Treichville hospital, lat. 5.293541, long. -4.003898). No authorisation was required for sampling in the first three sites. Authorisation was obtained for sampling in the Cocody and Treichville hospitals. The sites corresponded to open-air or closed sewage systems. Two of these were from geographically distant hospitals waste water purification stations. The different steps of phage isolation were described previously [26]. Briefly, filtrated water was incubated overnight in LB medium with enrichment bacteria, and, after centrifugation, the supernatant was spotted onto different bacterial strains including the enrichment strain. A total of 22 and nine phages were isolated in October 2010 and November 2011 respectively. Phage Ab12 which formed plaques on strain C5-2 in the first isolation step could not be further maintained in any tested strain. After two rounds of purification using a single plaque, phages were amplified on solid plate by infecting 200–400 μl of a 2x10^10^ cfu/ml bacterial suspension with one 24 hours old lysis plaque. One of the phages, Ab01, was previously described [26], and detailed analysis of Ab31 has been reported recently [36]. Phages were given names as recommended by Kropinski *et al*. [37].

### Host-range spectrum

Phage host range was determined by spotting 10 μl of purified phages with high titer (at least 10^9^ pfu/ml) on stationary *P*. *aeruginosa* cells. Presence or absence of a lysis zone was appreciated after overnight incubation at 37°C. Phage sensitivity was then confirmed using plaque assay. Briefly, 10 μl of undiluted phage suspension were directly streaked on LB agar plate, and 4 ml of molten top agar (0.7%) containing 2x10^9^ bacteria were poured over the phages. Characteristics of isolated plaques were recorded.

### Electron microscopy

Purified and concentrated phages were prepared and visualized by staining 5 μl of phage suspension with 2% potassium phosphotungstate (pH 7.0) as previously described [26].

### DNA purification and enzymatic digestion

DNA preparation was performed using a rapid DNA purification assay as previously described [38]. Briefly, 4 ml of amplified phage suspension were treated with 50 μg/ml RNase A for 30 min at room temperature, then 0.2 ml 2M Tris-HCl pH 7.5, 0.4 ml 0.5M EDTA, 0.2 ml 10% SDS and 10 μl diethylpyrocarbonate (1.1 g/ml, Sigma-Aldrich) were added. Following incubation at 65°C for 30 min, 1 ml 5M KOH was added, and the samples were left for 1 h on ice. Centrifugation was performed at 25,000 *g* for 20 min at 4°C, and the supernatant was precipitated with two volumes of ethanol. DNA was pelleted by centrifugation and washed twice with 70% ethanol then dissolved in 0.4 ml TE (10 mM Tris-HCl (pH7.5), 1 mM EDTA). Enzymatic digestion was carried out on 8 μl DNA in a final volume of 12 μl according to the manufacturer’s recommendations (Thermo Fisher Scientific, France), and analysed on a 0.8% agarose gel.

### Genome sequencing and analysis

Illumina paired-end sequencing was performed by BaseClear (Leiden, The Netherlands) or IMAGIF (Gif sur Yvette, France). The coverage was on the order of 1000X. The 100 bp reads were *de novo* assembled using Velvet [39] and Ray [40] as embedded in BioNumerics version 7.5 (Applied-Maths, Ghent, Belgium). Sub-samples of the initial read files were used, with 5000 to 100,000 reads, and several values of the k-mer length were tested in order to try and obtain a single contig. Once a single circular contig was obtained, the totality of reads was mapped against the sequence in order to identify regions with particular coverage, or the existence of ambiguity at some nucleotides, and to detect reads that did not map to the phage genome. In several phages, the presence of a high peak of reads which all stopped at the same position indicated the end point of a linear genome, as previously observed [24,26]. The genome starting point and the position of a putative Direct Terminal Repeat (DTR) were revealed by a particular distribution of the sequencing reads or by comparison with closely related phages. Multiple alignments were performed using the Geneious 8.1 software platform (Biomatters Ltd, Auckland, New Zealand) running MAFFT [41]. Annotation of open reading frames (ORF) was performed with RAST [42], using a 0.1 blastp E-value cut-off, and ARAGORN was applied to localize tRNA genes [43]. SNPs identification, Minimum spanning tree, and dN/dS analyses were performed in BioNumerics version 7.5 using the full bacteriophage sequences. Phage genome assemblies have been deposited to EBI/EMBL: Ab02 LN610572, Ab03 LN610573, Ab04 LN610581, Ab05 LN610574, Ab06 LN610582, Ab08 LN610575, Ab10 LN610586, Ab11 LN610583, Ab15 LN610587, Ab17 LN610576, Ab18 LN610577, Ab19 LN610584, Ab20 LN610585, Ab22 LN610578, Ab27 LN610579, Ab28 LN610589, Ab29 LN610588, Ab30 LN610590, 1-15_pyo_ LN610580.

## Results and Discussion

### Isolation of phages from sewage water

In order to favour the isolation of a variety of phages, we selected, in addition to the reference strains PAO1 and PA14, six clinical strains (C3-16, C7-6, C5-2, C8-14, C8-20 and C50) that were previously shown to be resistant to a large collection of phages of different genera (including those of the Pyo-Phage batch tested), and three clinical strains (C9-11, C2-10, SCH) with large susceptibility to these phages [26]. A spot assay was performed with the supernatant, and phages present in lysis zones were purified by several rounds of single plaque isolation. When different plaque morphologies were observed with a single sample, they were recovered and purified. Thereafter, a limited number of amplification strains were chosen for the good level of phage growth, PAO1 and C2-10 being most frequently used. The water sample origin, the date of isolation and the bacterial strains used to isolate and amplify new phages are summarized in Table 1. Interestingly, phage Ab22 produced both clear and turbid plaques on strain C2-10, in high density zones, and this phenotype was seen even after replating of a clear or a turbid plaque (S1 Fig). A similar phenotype was previously observed with phage p2-10_0r_, a LUZ24-like phage isolated in Orsay, France [26], and with other phages of this genus [44]. Phage Ab05 produced clear plaques on PA14 and PA01, and a halo was seen on PA14. Phage 1-15_pyo_, a ɸKMV-like phage isolated from Pyo-Phage and used here as a control, made similar plaques with a halo on PAO1 [26].

All phage genomes were digested with different restriction enzymes, showing that the banding pattern were identical or very similar in some phages, whereas others had specific profiles (data not shown). Combining the results of different enzymatic digestions, phages Ab05, Ab09, Ab22, Ab26, Ab30 and Ab31 appeared to be unique, while the other phages fell into five groups, including twelve, five, four, and three members respectively.

### Whole genome sequencing

Sequencing of all 30 phages was performed using the Illumina technology, and the reads were assembled producing a single contig. In some phages, upon alignment of reads against the assembled sequences, and in agreement with a previous observation for Ab01, high peaks of reads could be observed at two positions, delimitating a Direct Terminal Repeat (DTR) at the genome ends (S2 Fig). Assembled genomes were aligned with all available *P*. *aeruginosa* phage genomes, in order to identify the closest one. For all phages except Ab31 and the Ab18 group (Ab18, Ab19, Ab20, Ab21), 85–95% alignment was possible along the whole sequence of a known genome. The genome size of the new phages, and the genus they belong to, are indicated on Table 2. Some genome sequences appeared to be almost identical suggesting that the same phage was isolated several times independently. The 30 recovered phages correspond to 22 distinct phages. Interestingly, the GC content of most phages was largely inferior to that of the bacterial chromosome (66,6% for PAO1), except for phages of the YuA and DMS3 genera, and for Ab05. The putative ORFs were identified for each phage and annotation was performed by comparison with closely related genes. The proteins were categorized into different functional groups: morphogenesis and packaging, DNA replication, modification and recombination, regulatory functions, nucleotide synthesis. In total 21 among the 22 independent phages could be assigned to nine known genera, which are described below, and one phage, Ab31, was a combination between a virulent and temperate phage. The detailed analysis of Ab31 which showed limited homology at the DNA level with several Lambda-like phages, has been reported independently [36].

**Table 2:** Characteristics of the new phages.

Phage ID	Genome size (bp)	Family	Genus	Average similarity	Other similar phages^a^	DTR (bp)	tRNA	GC (%)
Ab01	92777				Ab07	1153	14	49,3
Ab02	93848					1165	14	49,4
Ab08	93503	Myovirus	PAK_P1	90%	Ab14, Ab16, Ab13	1165	14	49,2
Ab10	93053				Ab25	1165	14	49,3
Ab15	93308				Ab23, Ab24	1165	14	49,3
Ab03	86246					771	3	54,7
Ab04	86668					771	3	54,6
Ab06	84759	Myovirus	KPP10	90%		756	3	54,6
Ab11	85783					771	3	54,5
Ab17	83598					771	3	54,6
Ab27	66299					none	0	55,7
Ab29	66326	Myovirus	PB1	97%		none	0	55,6
Ab28	66181					none	0	54,9
Ab09	72028	Podovirus	N4	93%		641	0	54,9
Ab05	43639	Podovirus	ɸKMV	98%		431	0	62,3
Ab22	45808	Podovirus	LUZ24	91%		184	3	52,4
Ab18	56537					none	0	63,5
Ab19	58139	Siphovirus	YUA	70%	Ab21	none	0	63,3
Ab20	57745					none	0	63,5
Ab26	43055	Siphovirus	PA73	87%		none	0	53,4
Ab30	37238	Siphovirus	DMS3	95%		none	0	64,1
Ab31	45550	Podovirus	New			none	0	57,1

^a^ according to [30]

^b^ showing only a few SNPs

#### PAK_P1-like viruses

**Fig 1:**
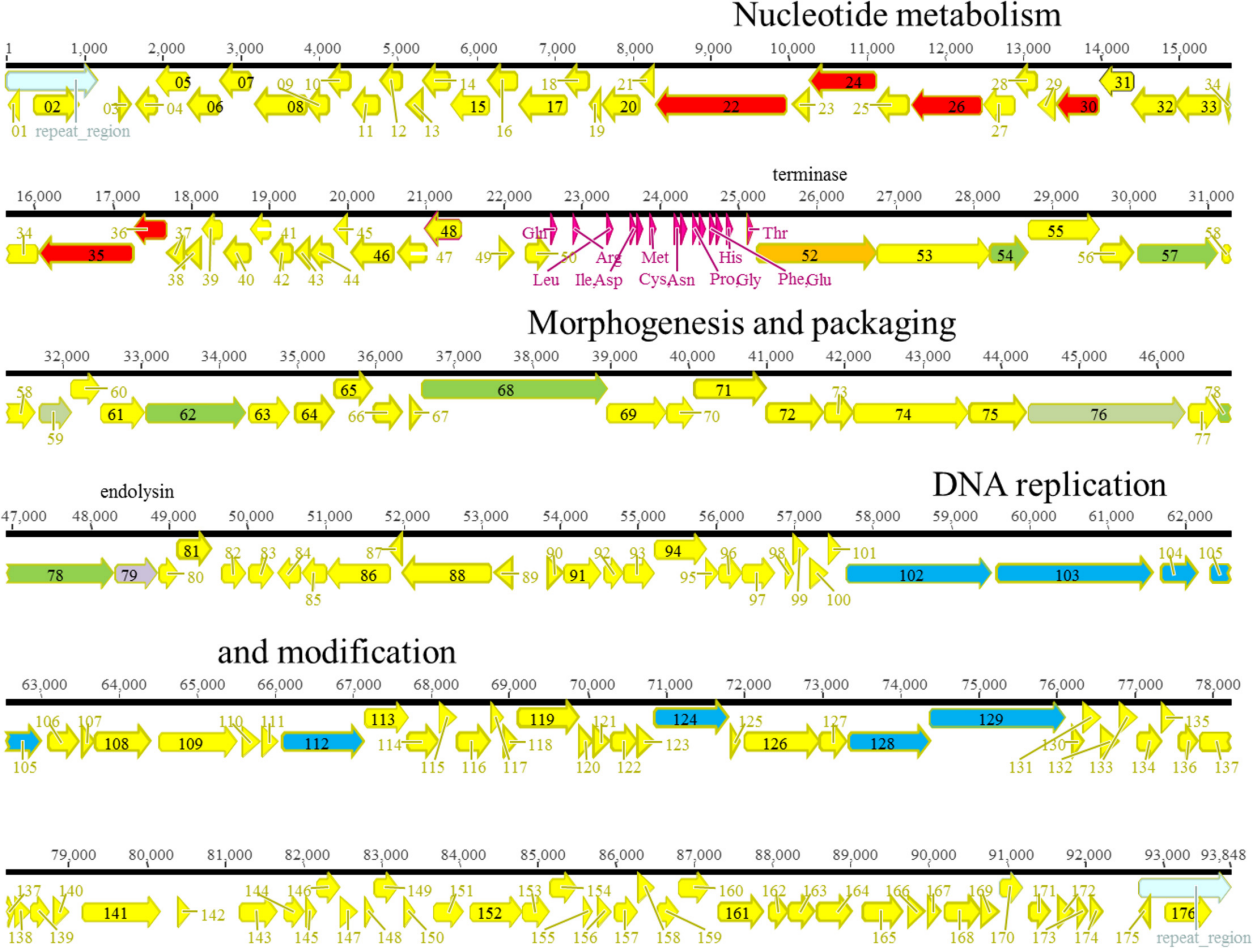
Genomic organization of PAK_P1-like phage Ab02. ORFs are shown as arrows. The different colors correspond to the putative function: yellow, unidentified, red, nucleotide metabolism, orange, terminase, green, morphogenesis and packaging, dark blue, DNA replication, light blue, DTR.

**Fig 2:**
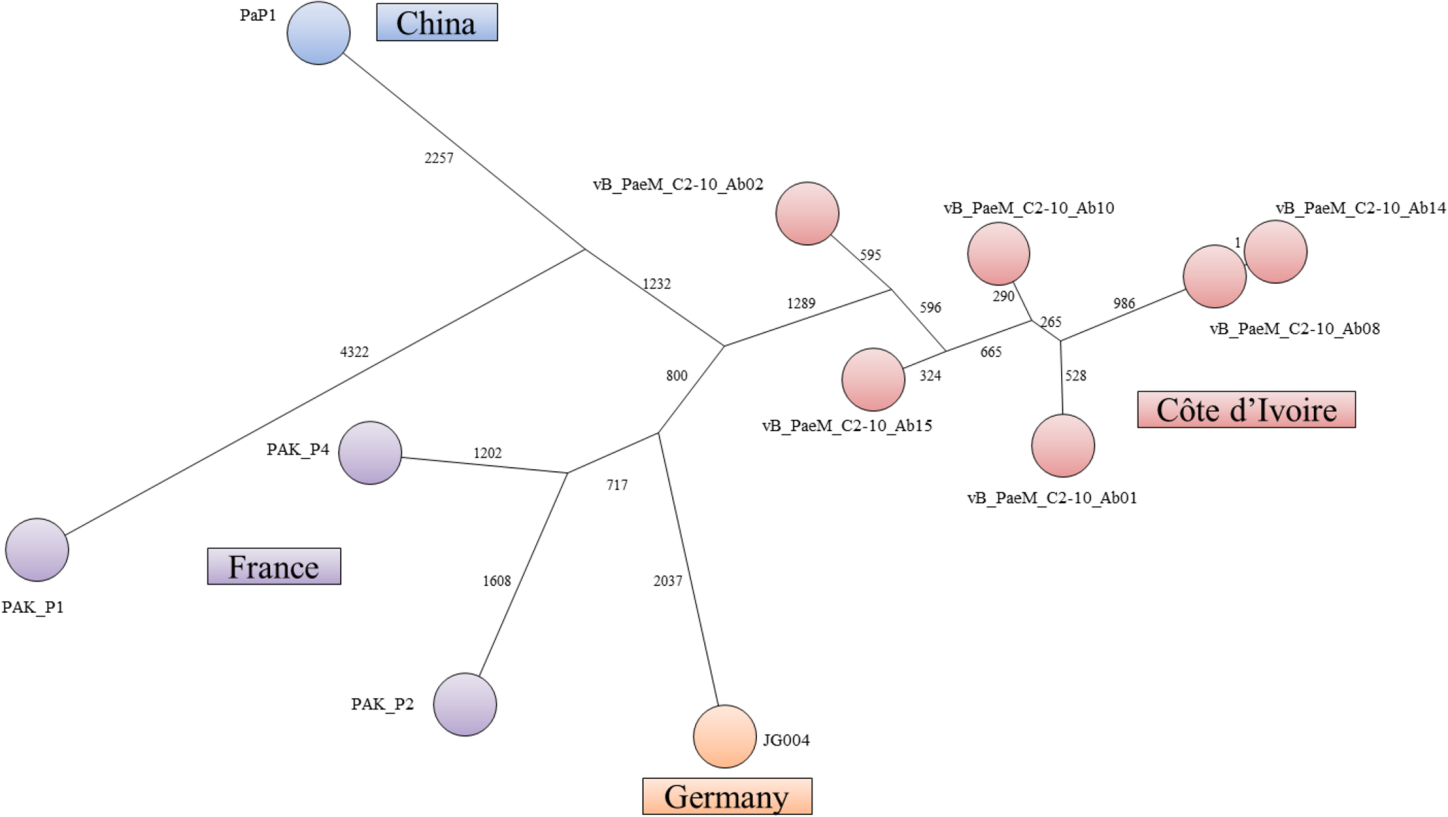
Minimum spanning tree representation of PAK_P1-like phages genomes. The numbers indicated on each branch represent the number of SNPs making this branch. A total of 12125 SNPs were identified and the tree size is 19714 indicating a high level of homoplasia. Homoplasia might result from independent HGT events with unknown phages infecting other *Pseudomonas* species. Colors indicate the phages country of origin.

The twelve phages from this virulent phage genus, including the previously reported phage Ab01 [26], were isolated at four different locations in Abidjan mostly in 2010 but also once in 2011. They were distributed into five different subgroups within which the genome sequences differed at only few nucleotides. They presented more than 90% similarity with the genomes of PAK_P1 from France [45], PAP1 from China [25] and JG004 from Germany [24]. For all twelve phages of this genus, DTRs of 1153 bp (Ab01, Ab07) or 1165bp (Ab02, Ab08, Ab10, Ab13, Ab14, Ab15, Ab16, Ab23, Ab24 and Ab25) were found, similarly to those observed on closely related PAK_P1-like genomes. The DTR size difference was due to insertion/deletions of a few base pairs. All genomes possessed 14 tRNA genes and coded for their own DNA polymerase, and for the control of nucleotide metabolism. Fig 1 shows the organization of ORFs on phage Ab02, and S1 Table lists the ORFs with a putative function. One genome of each subgroup was selected for multiple alignments revealing patches of high heterogeneity, but also several regions (3–9 kb) with only a few single nucleotide polymorphisms (SNPs) (S3 Fig). Patches of sequences showing a high level of divergence and low dN/dS values were observed in the first 17 kb of the genome, reflecting events of horizontal genetic transfer (HGT) [46]. By contrast some regions were devoid of traces of HGT and differed by a few SNPs and high dN/dS values. Presence/absence of sequences was noted. Ab08 possessed a gene encoding a putative endonuclease with an H-N-H motif (Ab08 ORF38, between ORF 39 and ORF40 of Ab02). The gene is absent from the other phages from Abidjan, but present in JG004 (PJG4_036) and PAK_P2 (00161c). In Ab02 a 759bp sequence encoding ORF 104 was inserted into the polymerase gene, separating it into two genes, encoding DNA polymerase part I (ORF103) and part II (ORF105). The insert was related to an intron described in the DNA polymerase gene of LUZ24 (PPLUZ24_gp35), and encoding its own endonuclease. Comparison with other phages of the same genus (JG004, PaP1 and PAK_P1) showed a higher level of diversity as reflected in a minimum spanning tree representation (Fig 2). The very high sequence similarity level in the African phages in regions of the genome not affected by HGT is in favour of a recent diversification from a common founder. A remarkable conservation of protein sequences was observed, such as for the major capsid protein which was identical in all the phages, as previously described [25]. A region of 827bp, present in JG004 only (position 34,438 to 35,264), encompassed the gene for an endonuclease (PJG4_070), inserted between the genes homologous to Ab02 ORF62 and ORF63.

A 21 bp polymorphism in the phages of this group corresponded to the length of a short duplication. It encoded a seven amino acid peptide (VGAPWYS), part of the hypothetical ORF75 of phage Ab02, containing a putative leucine zipper-like domain. Phages Ab02, Ab15, Ab24 and Ab25 had one copy whereas the other phages had two copies, and this polymorphism was confirmed by PCR amplification (data not shown).

#### KPP10-like viruses

**Fig 3:**
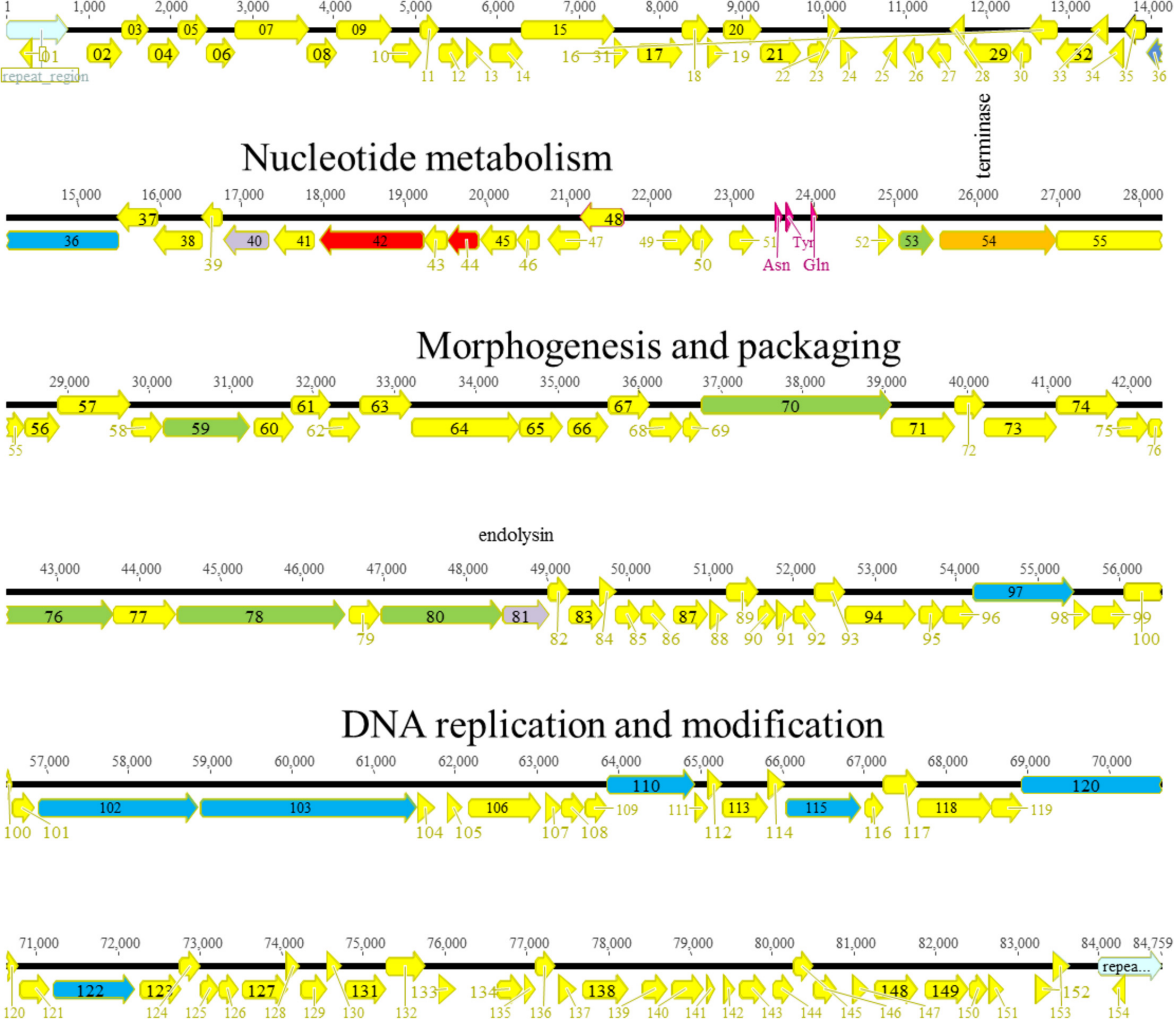
Genomic organization of KPP10-like phage Ab06. The different ORFs are colored according to their putative function: yellow, unknown; red, nucleotide metabolism; orange, terminase; green, morphogenesis; blue, DNA replication.

**Fig 4:**
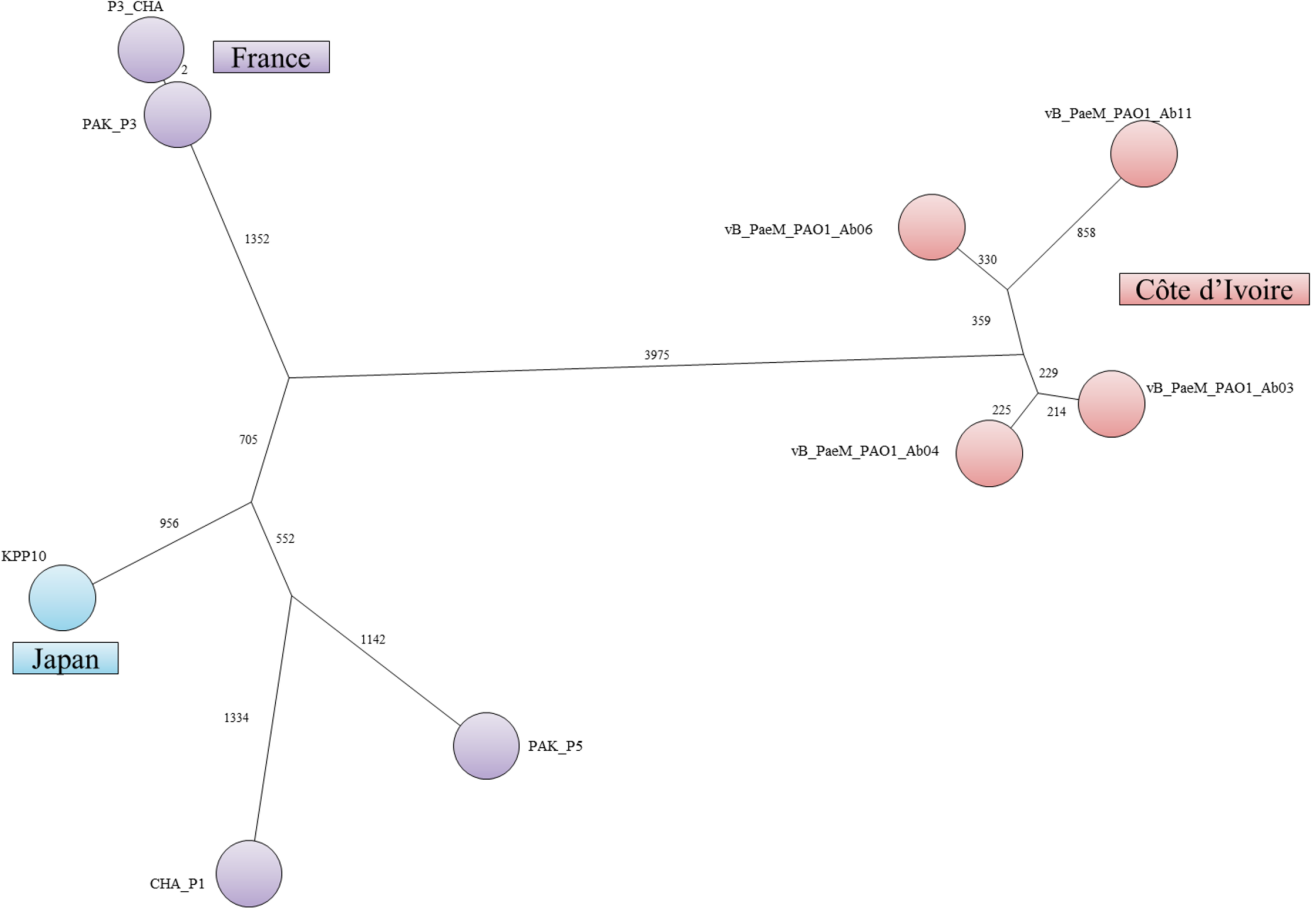
Minimum spanning tree representation of KPP10-like phages genome. The numbers indicated on each branch are the number of SNPs constituting this branch. A total of 9097 SNPs were identified and the tree size was 12233 indicating a significant level of homoplasia. Colors indicate the phages country of origin.

Five phages (Ab03, Ab04, Ab06, Ab11, Ab17) isolated in 2010 and 2011 at four different locations in Abidjan, belonged to the KPP10-like virulent phage genus. They were all different, and shared 88–96% sequence identity with the genomes of KPP10 from Japan [27], and PAK_P3 from France [23,45]. At position 23,000, a region with very low read coverage was observed in all phages except Ab17 and corresponded to an AT-rich region. A putative DTR of 771bp (Ab03, Ab04, Ab11, Ab17) showing an internal 15 bp deletion in Ab06, was found, defining the genome ends. The inferred nucleotide one position was different from the reported first nucleotide of phage KPP10. The genomes of the five phages were annotated (Ab06 is shown on Fig 3) showing that, overall, the ORF organization was very similar to that of the related phages, with a few differences for some very short hypothetical proteins. In keeping with KPP10, these phages had three tRNA genes (tRNA^Asn^, tRNA^Tyr^ and tRNA^Gln^). Upon alignment of the five genomes using Geneious, several regions of variability were observed within otherwise largely similar sequences (S4 Fig). In regions most probably encountering frequent HGTs, the percentage of SNPs was 20% as compared to 0.1% on average in the rest of the genome, and dN/dS ratios were low. Similarly to PAK_P1 phages, the first 22 kb, and the last 8kb showed the largest traces of HGT. It is interesting to note that some recombination sites appeared to be inside coding regions leading to the production of putative proteins with high levels of heterogeneity. This was the case for example for ORF14, encoding an hypothetical protein corresponding to ORF120 in KPP10, which was very different in the five phages, and appeared to be a patchwork of short regions from different origins. Similarly to PAK_P1-like phages, the five KPP10-like phages from Abidjan clustered at a large genetic distance to phages isolated in other countries (Fig 4). Several regions of short insertion/deletions were observed between the different phages, sometimes resulting in the fusion of two putative ORFs. Ab17 lacked a 3271 bp region encompassing seven hypothetical ORFs (ORF89 to ORF96 in Ab06), perfectly conserved in the other phages. As a result, a putative RNA ligase (ORF91 in Ab17) was formed by the fusion of the beginning of ORF89 and ORF97 (also a putative RNA ligase in the other phages) in Ab06. This region corresponded to nucleotides 22,874 to 26,077 in KPP10, and also encompassed seven putative genes of unknown function (ORF34 to ORF40). Another region of 528 bp was absent in Ab03 and Ab06, and corresponded to DNA with no homology in Genbank/EMBL at the nucleotide level.

#### PB1-like viruses

**Fig 5:**
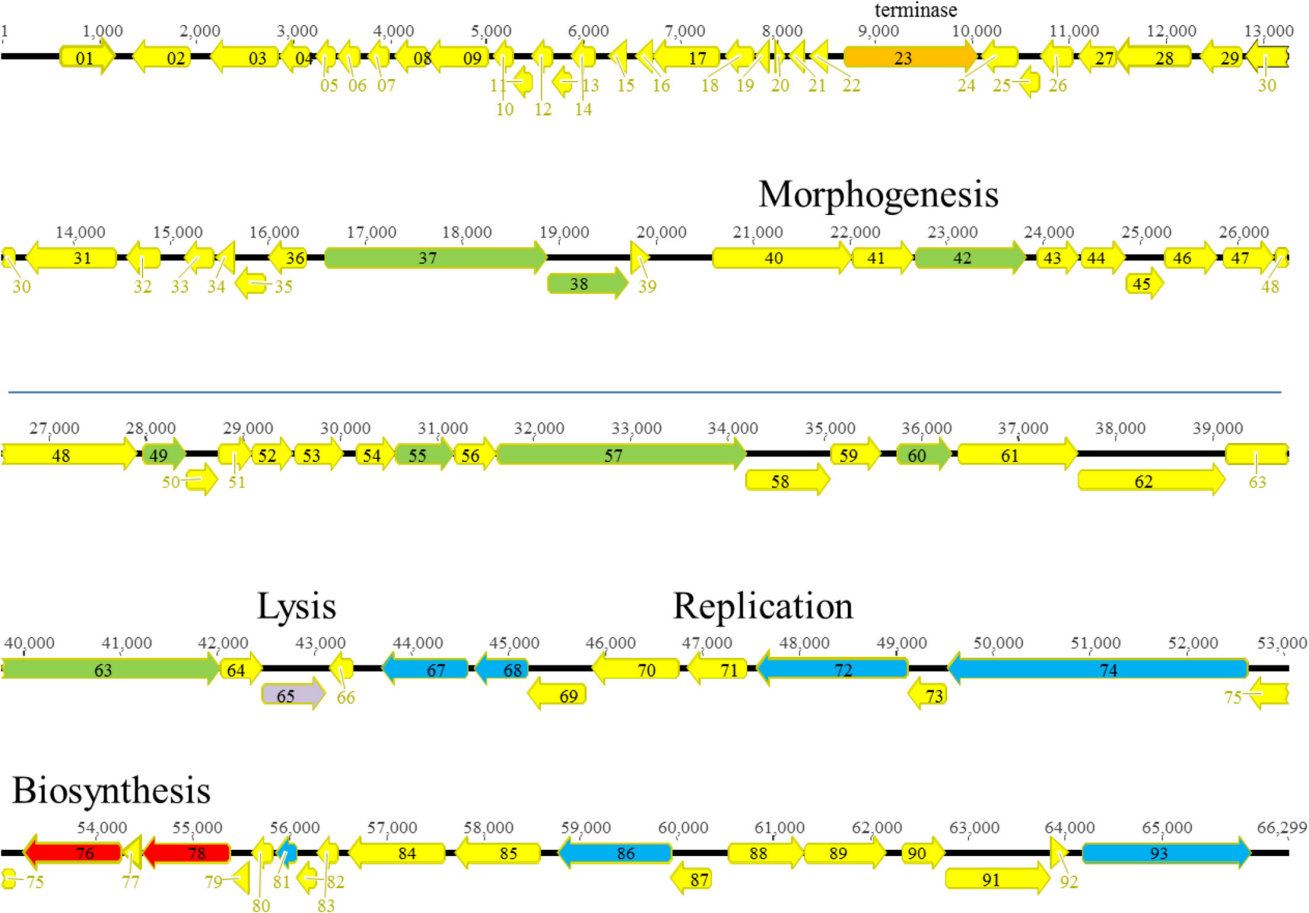
Genomic organization of PB1-like phage Ab27. The different ORFs are colored according to their putative function: yellow, unknown; red nucleotide metabolism; orange, terminase; green, morphogenesis; blue, DNA replication; purple, lysis.

Three phages (Ab27, Ab28 and Ab29) presented on average a 97% similarity level with several PB1-like virulent phages such as JG024 from Germany (66,275bp) [47], KPP12 from Japan (64,144bp) [48], NH-4 from Ireland (66,116 bp) [49], and SN from Russia (66,390bp) [50]. The presence of a category of reads with a single fixed termination tentatively marked the position of the phages genome ends, but there was no indication of a DTR. Based on this information, the first nucleotide could be positioned about 7,500bp upstream that reported for phage PB1. Alignment of the three phage genomes showed that Ab27 and Ab29 were quite similar, except for the first 4,400bp and the last 11,400bp where traces of recombination events could be seen (S5 Fig), characterized by a high density of nucleotides variations. Ab28 was very different from the other two at the nucleotide level, but the ORFs were remarkably well conserved in the three phages, and other PB1-like phages. The genome organization of phage Ab27 is shown on Fig 5. There was no RNA polymerase, but there seemed to be a complete DNA replication machinery as observed in other PB1-like phages. Between Ab27 ORF60 and ORF61 (position 36,360), reads contained either 9 or 10 G suggesting a possibility of phase variation at this site. Interestingly a 30 bp sequence (GATGCCCCGGCGAACCGGGGCGGGGTGGTT) at position 8,087–8,187 of the phage genome was present as a spacer in the Clustered Regularly Interspaced Region (CRISPR) of several *P*. *aeruginosa* genomes. This structure is part of an adaptive immune system believed to play a role in *P*. *aeruginosa* resistance to bacteriophages and plasmids [51], but is usually not associated with resistance to lytic phages. Several studies have shown that in *P*. *aeruginosa*, CRISPRs carry mostly sequences of temperate phages [26,51]. Our observation suggests that the CRISPR-Cas system may play a role in regulation of PB1-like phages infection.

#### N4-like virus

**Fig 6:**
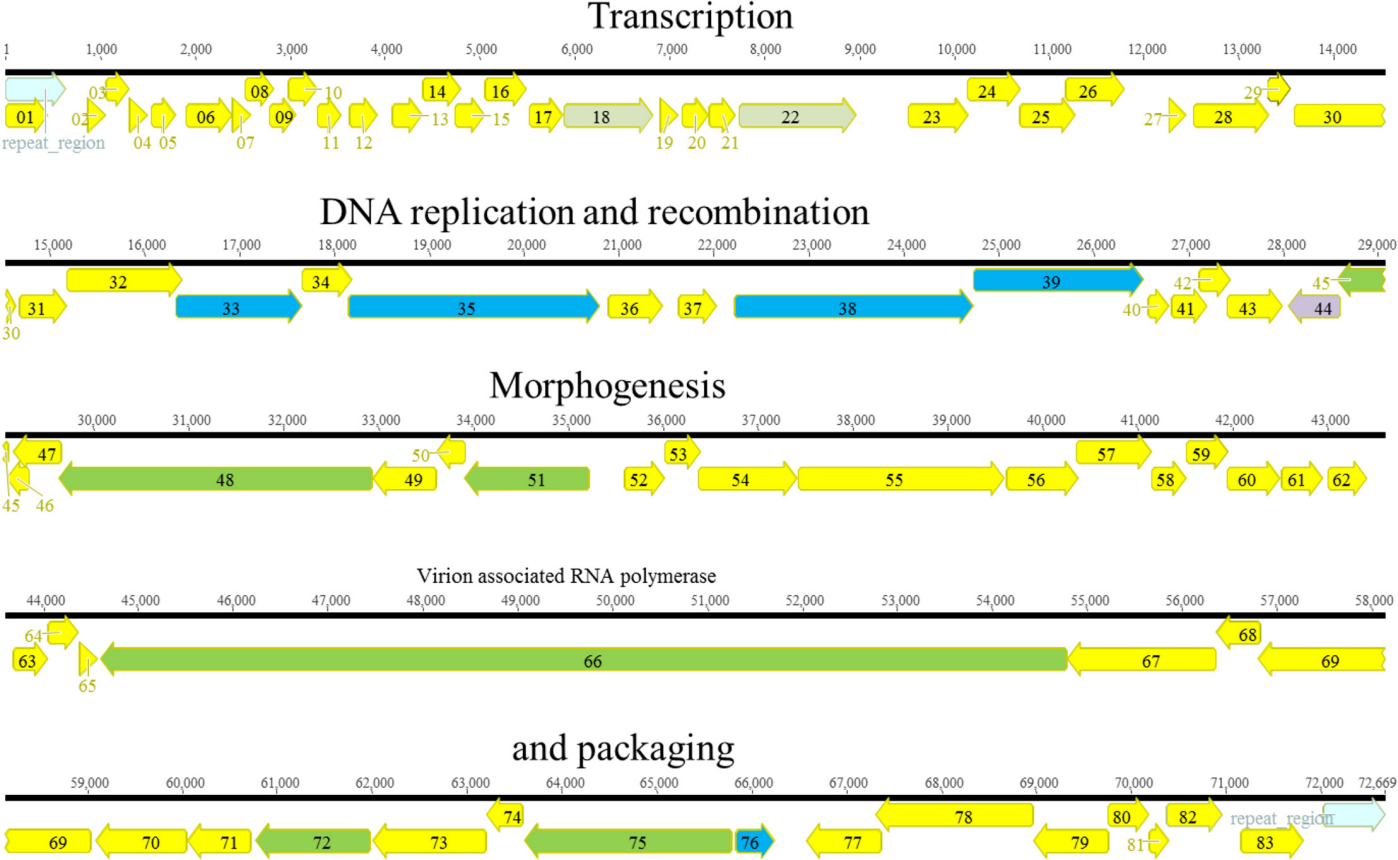
Genomic organization of N4-like phage Ab09. The different ORFs are colored according to their putative function: yellow, unknown; grey, transcription; green, morphogenesis; blue, DNA replication; purple, lysis.

The genome of Ab09 was 72,028 bp long. It showed a mean 93% similarity with lytic phage LIT1, a N4-like virus isolated in Belgium, (72,544 bp) [52], although some regions had less than 80% similarity with this phage. By contrast the mean similarity at the nucleotide level with phage LUZ7, another N4-like virus from Belgium, was only 65%. A 641bp DTR was found corresponding to the 655bp DTR of LIT1. The genome encoded 83 hypothetical proteins, among which a giant protein of 3398 amino acids (ORF66), the characteristic virion-encapsulated RNA polymerases of N4-like viruses (Fig 6) [52] which allows transcription of early genes in these phages. Ab09, like other N4–like phages encoded a second type of RNA polymerase (ORF18 and ORF19), a heterodimeric T7-like RNAP. Similarly to ORF 56 in phage LUZ7, Ab09 ORF48 aligned with ORF52 and ORF53 of LIT1, both putative tail proteins separated by 195 nucleotides [52]. No tRNA genes were identified. Similarly to other lytic phages with large genomes, a group of small hypothetical ORFs was found at one end of the genome.

#### ɸKMV-like viruses

**Fig 7:**
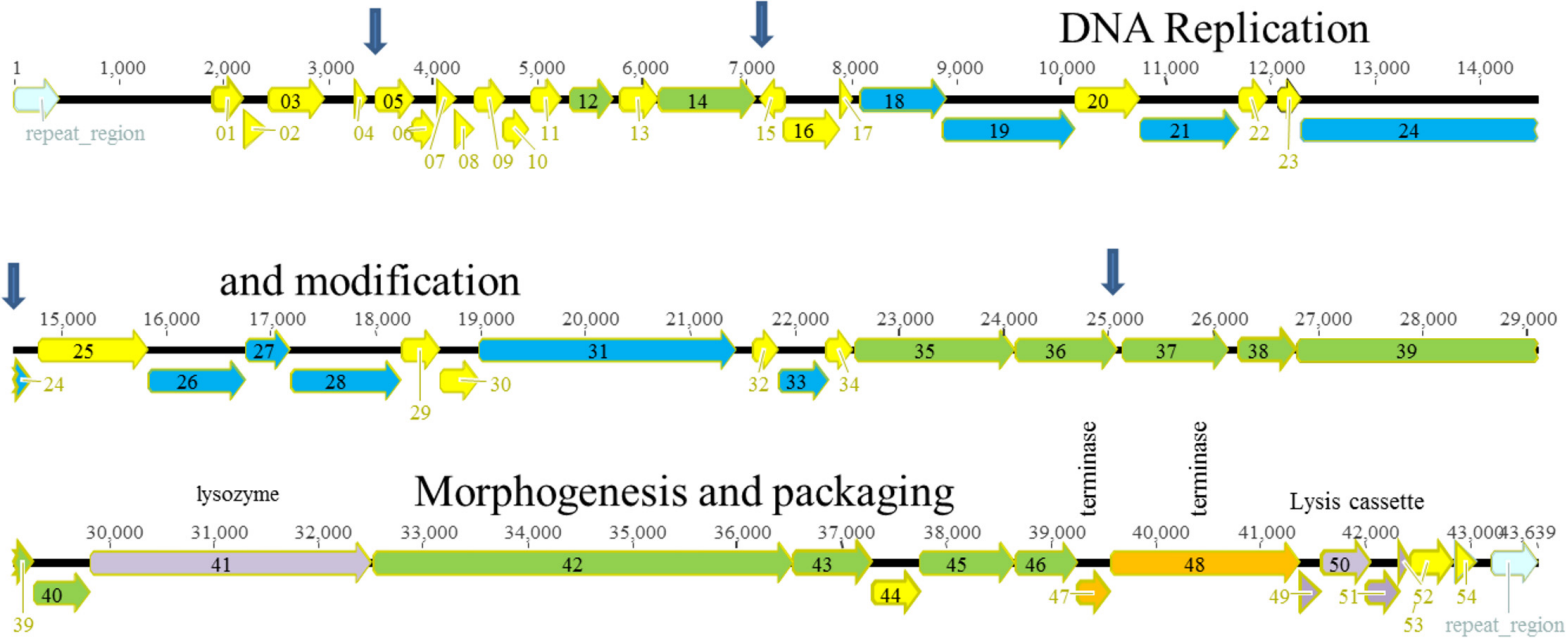
Genomic organization of ɸKMV-like phage Ab05. The different ORFs are colored according to their putative function: yellow, unknown; orange, terminase; green, morphogenesis; blue, DNA replication; purple, lysis. Vertical arrows indicate the position of single-strand DNA interruptions.

Ab05 (43,639bp) showed, on average, 98% sequence similarity with lytic phages LUZ19 from Belgium (43,548bp) [53], and ɸKMV from Russia (42,519bp) [54]. A 431bp DTR was observed, similar to that of LUZ19 (472 bp DTR). Overall the organization of putative genes was that of ɸKMV-like phages [10,55] (Fig 7). Contrarily to the large-genome viruses described above, Ab05 and the other ɸKMV-like genomes were more compact and essentially expressed genes for morphogenesis and replication, in addition to a group of small hypothetical ORFs. An RNA polymerase was found (ORF31), as typically observed in phages of this genus. The first genomic region ending after the gene for RNA polymerase, and encompassing genes for host conversion and DNA replication (early region), was the region showing the most diversity [55]. A lysis cassette formed of a pinholin (ORF49), endolysin (ORF50), and spanins (ORFs 51–52) was similar to the one described in ɸKMV-like phages [56]. Interestingly no putative ORFs were found in the first 1900 nucleotides, a region holding three to five strong promoters in other ɸKMV-like phages. The canonical nucleotide sequence 5’-CGACXXXXXCCTACTCCGG-3’, localized at putative sites for single-strand DNA interruptions [57], was found three times in the Ab05 genome (arrows on Fig 7). Ab05 showed, in addition, the variant sequence 5’-GGGCXXXXXCCTACTCCGG-3’. At these positions an excess of sequencing reads could be observed. Compared to other ɸKMV-like phage genomes, four deletions of putative genes, as well as many regions with low level of similarity at the nucleotide level were observed, reflecting recombination events. The two smaller deletions corresponded in phage LKD6 to a region containing a putative promoter and to an intergenic sequence [55]. Deletion 3 encompassed the short ORF17.1 of phiKF [58]. Deletion 4 encompassed gp20 in LUZ19, a short ORF present in all the sequenced ɸKMV-like phages.

#### LUZ24-like virus

**Fig 8:**
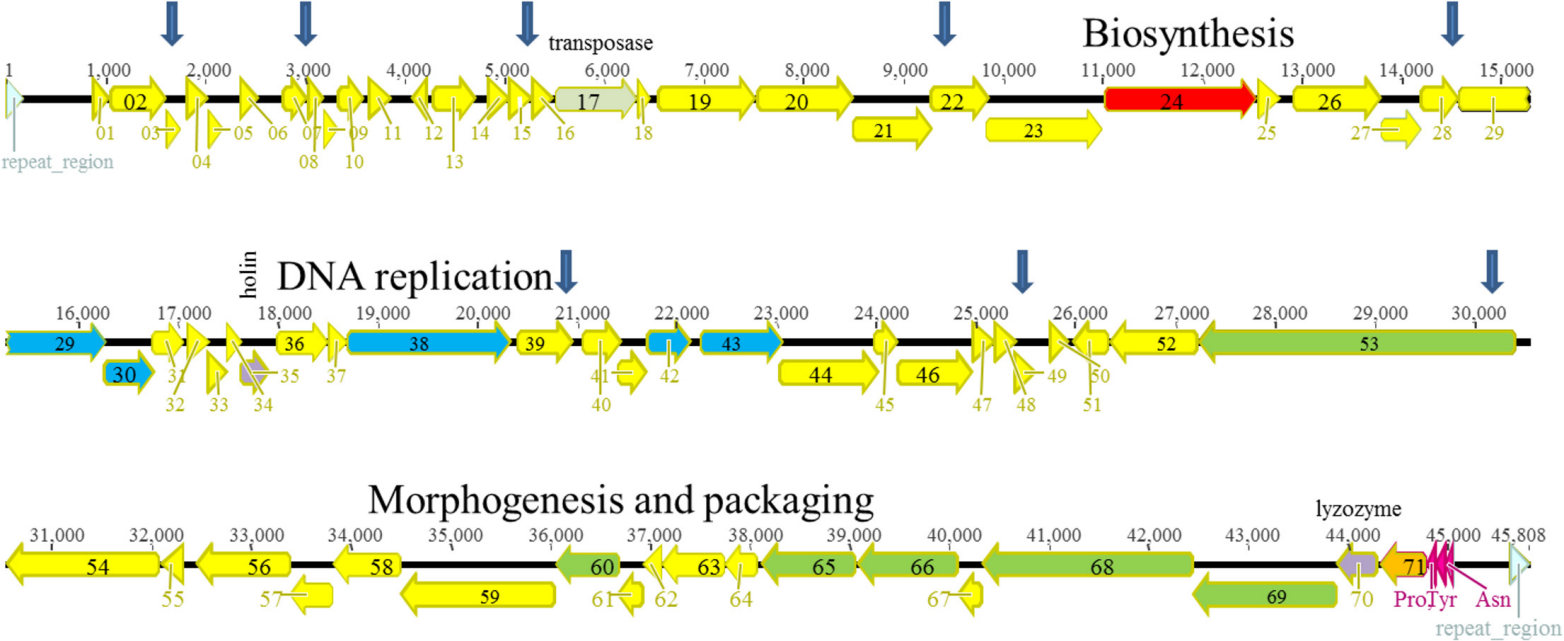
Genomic organization of LUZ24-like phage Ab22. The different ORFs are colored according to their putative function: yellow, unknown; red, biosynthesis; green, morphogenesis; blue, DNA replication; purple, lysis. Vertical arrows indicate the position of single-strand DNA interruptions.

The Ab22 genome was 45,808 bp long and showed 86–96% similarity with lytic phage LUZ24 from Belgium (45,625 bp; AM910650 [59]) and lysogenic phage PaP3 from China (45,503bp NC_004466 [60]). The genome was also close to that of phage 1-14_Or01_ that we previously isolated in France [26]. Ab22 possessed a 184 bp DTR as observed for 1-14_Or01_ (182bp) [26]. Annotation predicted the existence of 71 putative ORFs and three tRNAs (tRNA^Pro^, tRNA^Tyr^ and tRNA^Asn^) (Fig 8). By comparison, LUZ24 showed 74 ORFs and two tRNAs, and PaP3 71 ORFs and 4 tRNAs. Several regions of insertion or deletion with respect to LUZ24 were observed. The longest (1206 bp) was present in Ab22 (ORF17) and absent in LUZ24, and all other closely related *P*. *aeruginosa* phages. It showed 100% identity with the transposase fusion protein of phage TL from Russia (YP_009007804), suggesting that this gene possibly contributes to the insertion of the phage genome in the bacterial DNA. The second largest region of difference was a 665 bp fragment, absent in Ab22, encoding the gp35 endonuclease (self-splicing intron) in LUZ24, separating the polymerase part II and III (ORF34 and ORF36). These two genes were fused into a single ORF in Ab22 (ORF38). The first 1000 nucleotides did not encode any putative protein and probably contained promoters, although the consensus sequence described by Ceyssens et al. [59] could not be found at this position. At six positions (arrows on Fig 8), an excess number of reads corresponded to the sequence 5’-GTACTATGAC-3’, or to the variant 5’-GTACTGTGAC-3’ marking the single-strand DNA interruptions observed on the viral genome. We and others previously reported the existence of such sites with phages of LUZ24-family [26,61].

#### YuA-like viruses

**Fig 9:**
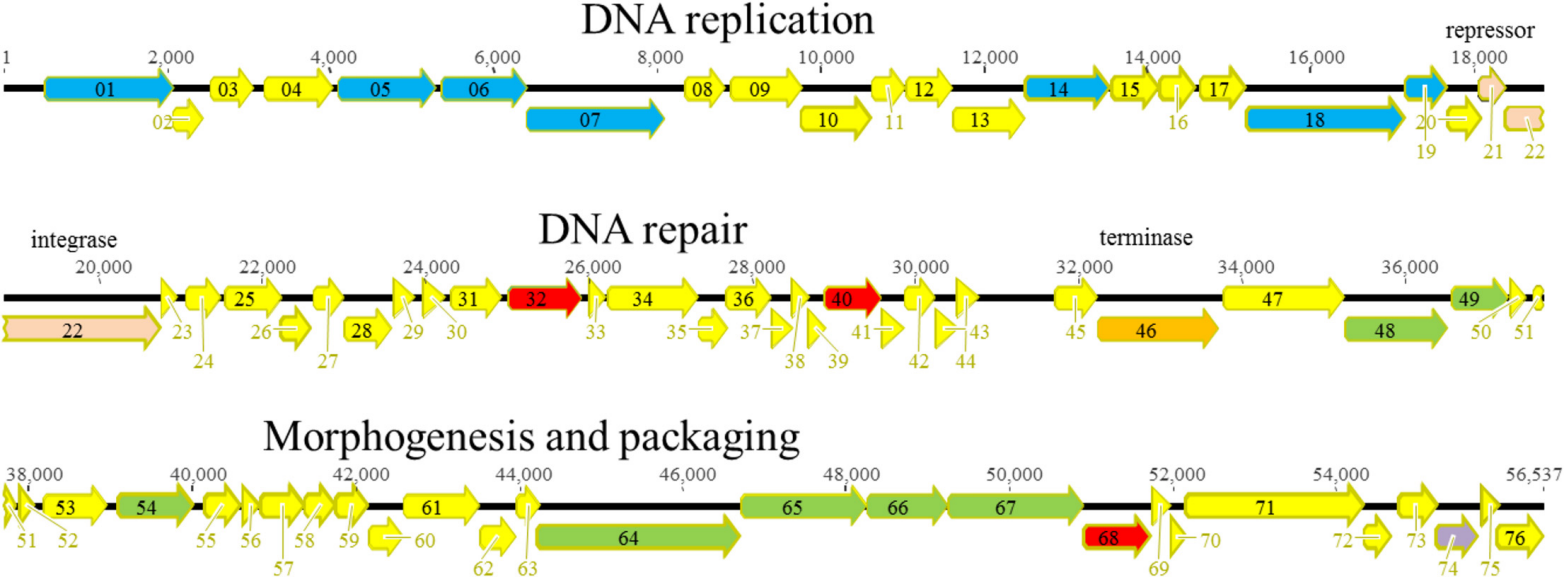
Genomic organization of YuA-like phage Ab18. The different ORFs are colored according to their putative function: yellow, unknown; orange, terminase; red, DNA repair; green, morphogenesis; blue, DNA replication; purple, lysis, pink, prophage insertion.

Ab18, Ab19, Ab20 and Ab21, isolated in 2010 and 2011 at two locations and showing related restriction profiles, appeared to be YuA-like phages. Genome sequencing showed that there were in fact only three different phages, Ab19 and Ab21 being identical. The sequencing reads aligned as a circular genome, with no abnormal peaks of reads and consequently the first nucleotide was assigned by comparison with the closest genome, YuA from Russia (58,663 bp) [62]. The genome of the three phages showed at best 70% similarity with that of YuA and MP1412 from South Korea (61,167 bp) [63]. At the protein level, additional similarities could be observed particularly in structural proteins. Moreover, the DNA polymerase (ORF18) and the terminase gene (ORF46) also showed an elevated degree of homology with those of YuA. Fig 9 shows the organisation of the 76 Ab18 ORFs, all oriented in the same direction. Overall the organisation was that of YuA with some remarkable differences, particularly at the level of small ORFs of unknown function. ORF6 and ORF7 of Ab18 and Ab19 encoded the small and large subunits of a ribonuclease reductase of class Ia which corresponded to a single gene in YuA. A putative repressor (ORF21) and an integrase (ORF22) showing 55% identity to that of YuA were present, suggesting that the phage could possibly lysogenize its host. Other parts of the genome had no homology with any phage in the public databases, either at nucleotide or protein level. Ab18, Ab19 and Ab20 genomes showed an average 95% similarity with each other, and displayed several regions of insertion/deletion. The genomes were aligned showing that overall the SNPs were evenly distributed, except for one large region of low percentage of similarity encompassing genes for the tail fiber proteins (S6 Fig). This is in contrast with the high level of divergence observed with the closest phage YuA, and provides a direct estimate of the relative role of mutation by descent versus recombination in this homogenous group of phages. YuA-like phage genomes were reported to be resistant to many restriction enzymes including *Eco*RI, although *Eco*RI restriction sites exist in their genome [7,62]. We observed a similar resistance in the four phages from Abidjan suggesting the existence of DNA modifications.

#### PA73-like virus

**Fig 10:**
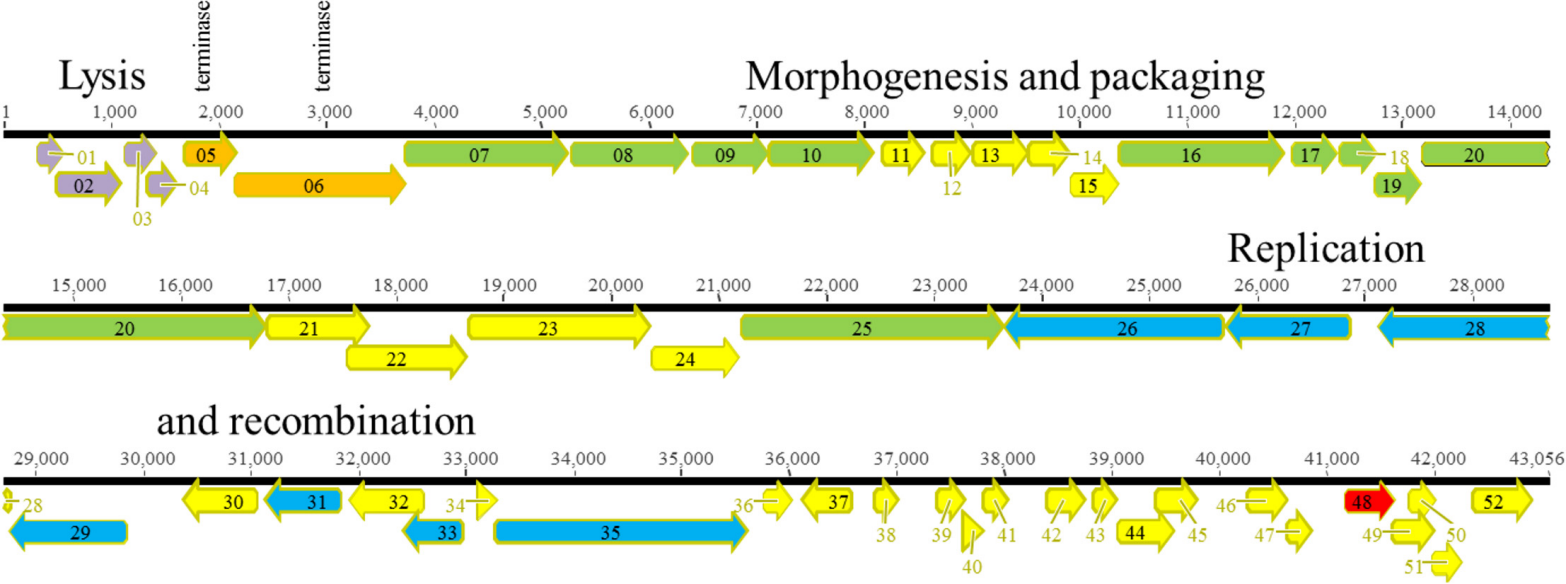
Genomic organization of PA73-like phage Ab26. The different ORFs are colored according to their putative function: yellow, unknown; orange, terminase; red, biosynthesis; green, morphogenesis; blue, DNA replication; purple, lysis.

Ab26 genome was 43,055 bp long and encoded 52 putative proteins (Fig 10). It showed 87% homology with vB_Pae-Kakheti25 from Georgia (42,844 bp; NC 007806) considered a lytic phage [64] and with PA73 from the Lindberg set (42,999 bp; DQ163913) [65]. The phage ends and the orientation of the genome map were aligned to that of related phages, and alignment showed a high overall conservation of gene organization between these phages except for three regions. The 296 bp at the beginning of the genome, encoding a protein found in a lysis cassette in phage vB_Pae-Kakheti25, were very different in the three phages. Interestingly, Ab26 possessed well conserved holin, endolysin, Rz and Rz1 spanin genes (ORF01 to ORF04) forming a lysis cassette similar to that described in ɸKMV-like phages [56]. One region of 1,600 bp encoding proteins found in mature virions (ORF21-ORF22), showed about 70% similarity with vB_Pae-Kakheti25 but 95% with PA73. Finally, from position 36,000, after the gene encoding a putative primase/helicase (ORF35), to the end of the genome a series a short hypothetical ORFs was observed showing a high degree of divergence as compared to the two closest phages. No integrase or other protein that could be involved in lysogeny were clearly identified. However ORF31, a recA-like recombinase, was shown to belong to the sak4 family of proteins, almost exclusively associated with temperate phages [66].

#### D3112/B3-like virus

**Fig 11:**
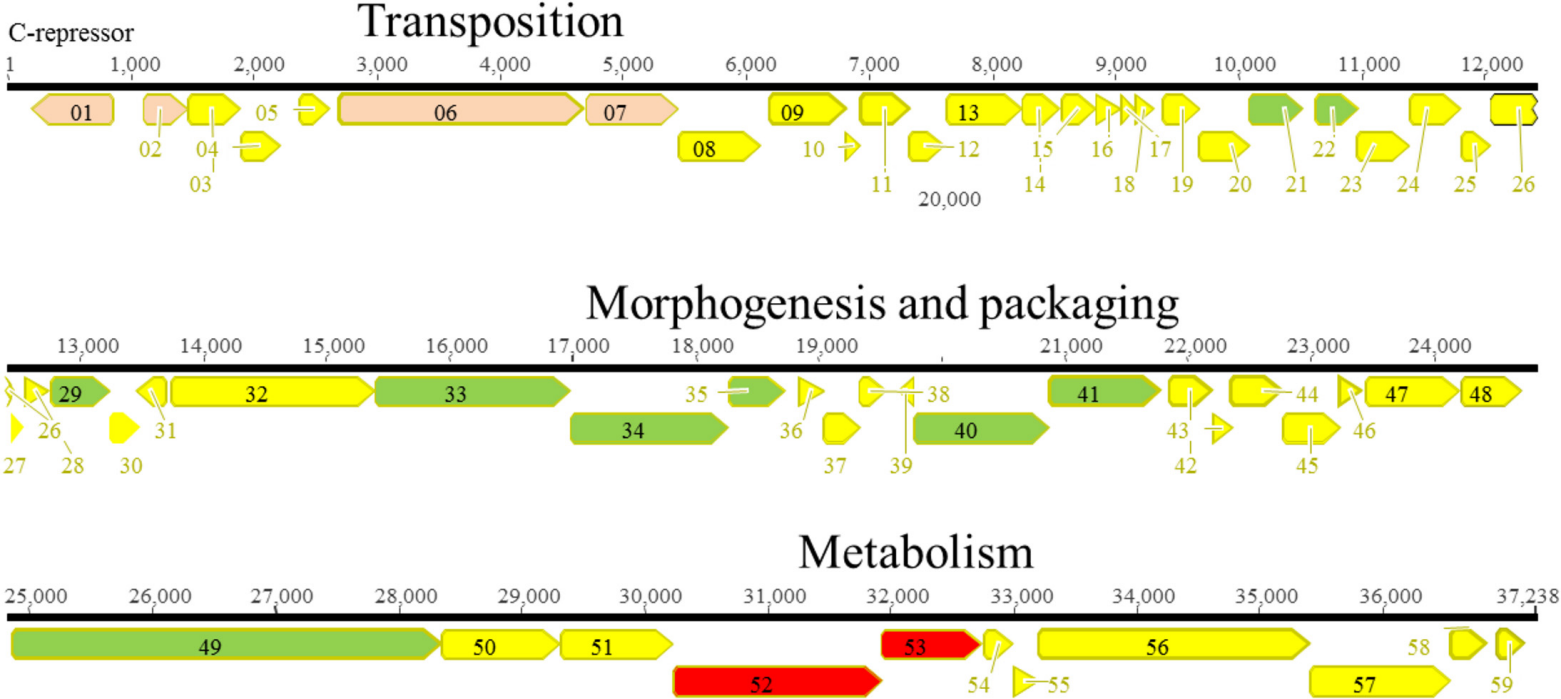
Genomic organization of D3112/B3-like phage Ab30. The different ORFs are colored according to their putative function: yellow, unknown; red, metabolism; green, morphogenesis; pink, transposition.

Ab30 had a 37,238 bp genome and showed between 91 to 99% similarity with transposable phage DMS3 from the USA (36,415 bp) [67], and phages MP38 (36,885bp; EU272037) and D3112 from Russia (37,611bp) [68]. These phages are related to the *Escherichia coli* phage Mu which replicates by transposition [69] and possesses chromosomal DNA fragments of variable size at the end of its genome. The presence of a characteristic c repressor gene (ORF01) and of transposases A (ORF06) and B (ORF07) genes in the early genome region suggested a similar mechanism of lytic-lysogenic switch (Fig 11). Similarly to other phages of this genus, Ab30 possessed an extensive mosaic structure but the gene organization was well preserved. Alignment with phage DMS3 revealed regions of high diversity such as ORF01, and additional genes encoding short hypothetical proteins. Previously, it was shown that inhibition of biofilm formation as well as swarming motility of DMS3-lysogenic bacteria was mediated by the CRISPRs-Cas system [67]. Atypical genes able to inactivate bacterial CRISPR-Cas system were identified within Mu-like phage genomes [70,71]. By comparison with published sequences of Mu-like phages, the group of hypothetical genes encoding ORF36 to ORF39 may represent an anti-CRISPR region.

### Virion structure

**Fig 12:**
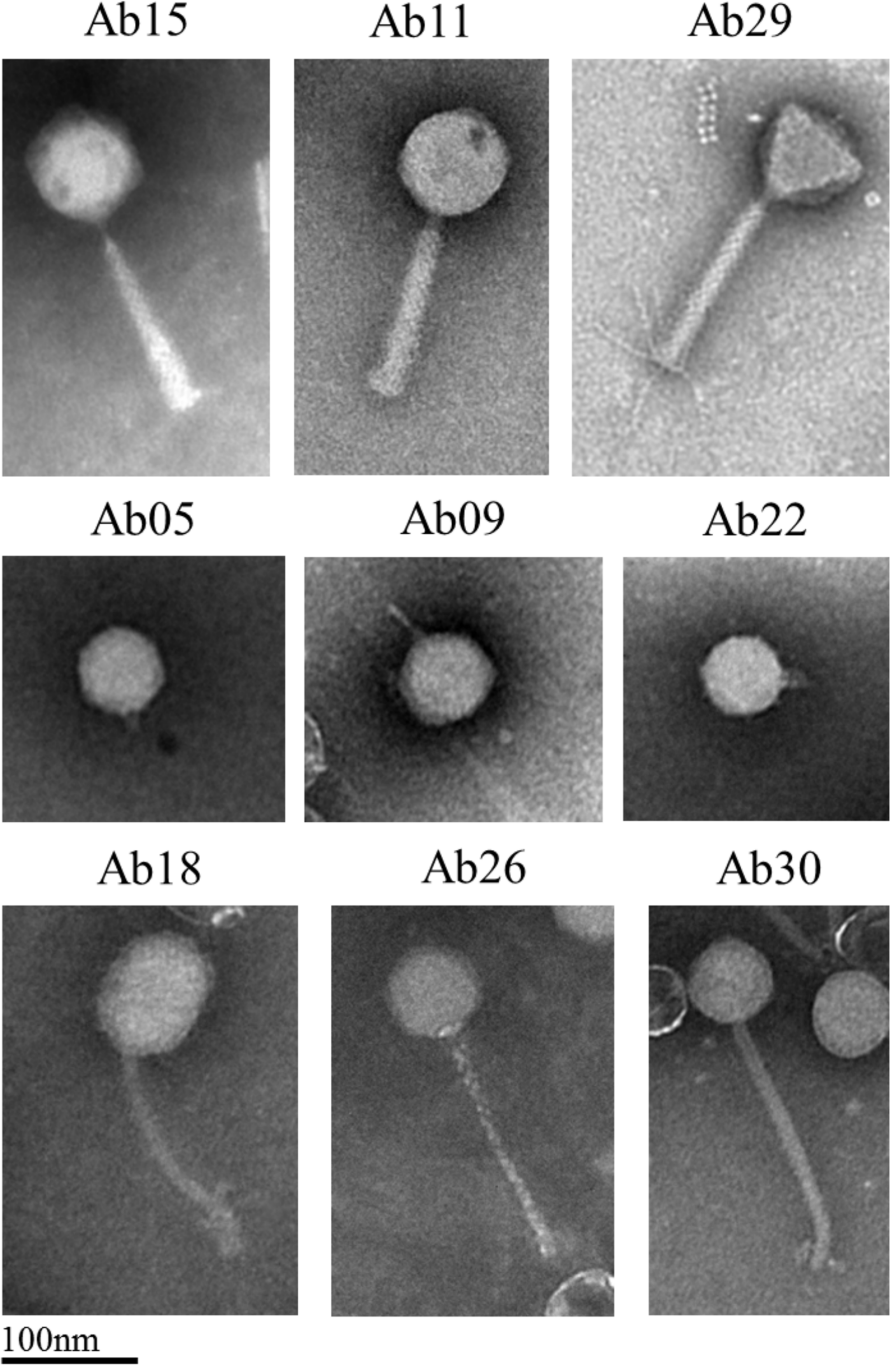
Electron microscopy examination of nine phages representative of the different genera observed in the present phage collection. Scale bar represents 100 nm.

EM examination was performed for at least one phage of each genus (Fig 12). As expected from the genome sequence, phages belonging to the PAK-P1, KPP10 and PB1 genera were myoviruses (A1 morphotype) with long contractile tails. Five PAK-P1-like phages were analysed and all showed the same morphology, with a 130 nm tail and a 67–70 nm head. Ab11, a KPP10-like phage, had a 70 nm head and a 120nm long tail, while virion particles of Ab29, a PB1-like phage, possessed a 74 nm head and a 140 nm long tail. Among podoviruses with short tails (C1 morphotype), Ab05 and Ab22, belonging to the ɸKMV-like and LUZ24-like genera respectively, displayed 60nm icosahedral heads, while Ab09, a N4-like phage, had a 70 nm icosahedral head. Ab18, Ab26 and Ab30 were siphoviruses with a B1 morphotype. Ab18 showed an elongated 85×60nm head and a 130 nm tail similar to that of phage YuA. Ab26 had a head of 60 nm and tail of 170 nm, Ab30 a head of 55 nm and tail of 180 nm.

### Host-range spectrum

Phage host range was determined on the collection of *P*. *aeruginosa* strains used to isolate the phages of this study, and on additional strains selected for their resistance to Pyo-Phage [26]. The complete result is shown in S2 Table, and Table 3 shows the virulence spectrum of one phage for each genus and that of four phages isolated from Pyo-Phage (p1-14_pyo_, p8-13_pyo_, p1-15_pyo_ and p2-10_pyo_). The members of a genus generally showed similar virulence towards the selected panel of bacterial strains with some exceptions. Together the present collection of lytic phages (all the myoviruses and the podoviruses except Ab31) which belong to six genera, could lyse 16 out of 20 tested strains. Four strains, C5-13, C8-14, C8-15 and C8-20 were resistant to all phages. Phages of the PAK_P1 genus showed the highest virulence both in term of spectrum and efficiency of plating. Seven of them showed a high plating efficiency on C7-6, a Pyo-Phage-resistant strain, in which they produced large clear plaques. On the same strain, Ab01, Ab15 and Ab23 showed a ten times lower growth, and Ab24 and Ab25 did not grow. No clear plaques could be observed on C7-6 with any phage belonging to the other genera. Similarly, most phages of the PAK_P1 genus could lyse two other Pyo-Phage-resistant strains, C8-5 and C8-7. The three PB1-like phages displayed a large host range, infecting Pyo-Phage-resistant strains C7-12, C7-25, C9-5 and C9-6, but with a lower efficiency as compared to growth on PAO1, SCH and C3-16. This resembled the broad host-range of related T4-like viruses. As previously shown [72], KPP10-like phages were very specific toward certain clinical strains, and displayed strong virulent activity on strain PAO1 on which clear plaques were observed. They produced turbid plaques in a limited number of other strains. Several members of lytic PAK_P1, PB1 and KPP10-like phages could be obtained, possibly reflecting, for the first two genera, their wide host range. However it is more surprising for the KPP10-like phages which efficient growth is restricted to PAO1 in our collection of strains. This might suggest that *P*. *aeruginosa* is not the preferred host for this genus. Ab22, a LUZ-24-like phage, produced clear and turbid plaques in strain C2-10 (Fig 1), and uniform turbid small plaques in strain PAO1. The host-range of ɸKMV-like phage Ab05 was similar to that of phage p1-15_pyo_, another ɸKMV-like phage, but with different efficiency, Ab05 being clearly less virulent. In particular p1-15_pyo_ produced large plaques with a halo characteristic of this genus in strain PAO1 [10], whereas Ab05 produced plaques with a small halo, only on PA14. The N4-like phage Ab09 had a rather large virulence spectrum but different from that of PAK_P1-like phages. It formed clear 1-2mm plaques on PAO1 without a halo, as opposed to other *P*. *aeruginosa* N4-like phages [52].

**Table 3:** Host-range of representative Abidjan and Pyo-Phage-derived phages.

Genus	PAK-P1	KPP10	PB1		N4		ɸ-KMV		LUZ24		YUA	PA73	D3112
Phages Bacteria	Ab08	Ab06	Ab27	p1-14_pyo_	Ab09	p8-13_pyo_	Ab05	1-15_pyo_	Ab22	p2-10_pyo_	Ab18	Ab26	Ab30
PAO1	-	c+	c+		c+	c+	c+	c+	t		c+	-	t
PA14	t	-	t	c+	-		c+	-	-	t	-	-	c+
Tr60	-	-	-	c+	-		t	t	t	c+	-	-	-
C50	-	-	-	-	-	-	-	-	-	-	c	-	t
SCH	c+	-	c+		c+		-	c+	-		c	c+	t
C1-1	-	t	c	t	c+	c+	t	c+	-		t	-	-
C1-2	c	t	-	t	c+	t	-	-	t	t	c	-	-
C1-14	c+	t	c+	c+	c+	c+	t	c+	t	t	t	-	-
C2-10	c+	-	t	-	c+	-	-	c	c+	c+	-	c	-
C3-16*	-	-	c+	-	t	c+	-	t	t	-	-	-	t
C3-20	-	-	-	-	-	t	-	-	c	c+	-	-	-
C5-2	-	-	t	-	c+	-	-	c+	-	-	t	t	t
C5-12	t	-	c	t	-	-	-	-	c	t	c	-	-
C5-13	-	-	-	-	-	-	-	-	-	-	-	-	-
C7-6	c+	-	-	-	-	-	t	t	-	-	t	-	-
C7-12	-	-	c	-	-	-	-	-	-	-	-	-	-
C7-25°	-	-	c	-	-	-	-	-	-	-	-	-	-
C8-5	c+	-	t	-	-	t	-	t	t	t	t	t	t
C8-7*	c	-	t	-	-	-	-	-	t	-	-	-	t
C8-14	-	-	-	-	-	-	-	-	-	-	-	-	-
C8-15	-	-	-	-	-	-	-	-	-	-	-	-	-
C8-20*	-	-	-	-	-	-	-	-	-	-	-	-	-
C9-5	c	-	c+	-	-	-	-	c	t	-	c	t	-
C9-6	c	-	c	-	-	-	t	-	-	-	-	-	-
C9-11	c+	t	t	c+	c+	c+	-	t	t	-	c	t	t
C9-17	c+	t	t	t	t	t	-	t	t	t	c	t	t

* slow growing strain

°continuous release of prophage

c: clear plaque; c+: maximum growth; t: turbid plaques

The siphoviruses of *P*. *aeruginosa* are majoritarily temperate phages and we expected them to display a rather specific host range. The group of phages related to YuA (Ab18, Ab19-Ab21, Ab20) showed a variety of virulence profiles, but the highest efficiency of plating was seen with PAO1 on which they produced small clear plaques (0.5–1mm). Interestingly only phages of this group infected the widespread European strain C50 (reference strain for clone C) that is also resistant to pyophage. Ab26 grew efficiently only on strain SCH and produced a more limited growth on several other strains. Phage vB_Pae-Kakheti25, belonging to the same genus possesses a very large host range toward clinical strains [64]. Ab30 showed a lytic activity only on PA14.

The ability of phages from the three siphovirus groups to form lysogens was tested by the presence of viral DNA by PCR in resistant colonies recovered from the centre of turbid plaques, and then passaged at least three times. Lysogens for Ab30 could be obtained in strain Tr60, as suggested by the stable presence of phage genome, and by mytomycin C induction of virions. Lysogens for Ab18 could not be obtained in strain PAO1, the most susceptible strain for this phage, as reported for YUA, and despite the presence of an integrase gene in the phage genome. In strain C2-18 in which only moderate growth was observed, the Ab18 DNA was maintained for several generations but disappeared upon further replating, suggesting a pseudolysogenic state. Ab26 genomes could be detected in resistant variants of SCH, the strain which supports its growth, but no stable lysogens could be obtained.

By mixing one member of each genus in the present collection, we were capable of lysing all *P*. *aeruginosa* isolates except four that appear to be also resistant to ɸKZ-like viruses [26]. Host range is dependent on interactions between the phage tail fibers and bacterial receptor, but additional mechanisms are involved in bacterial susceptibility to phages. Phage infection failure may be due to exclusion of superinfection as the majority of strains isolated from CF patients possess one or several prophages [26]. Indeed, the presence of prophages in a bacterial chromosome immunizes bacteria against infection by phages of the same nature [73]. During lysogeny, D3-like phages modify the LPS receptors, making bacteria resistant to LPS-dependent phages, including virulent phages [74]. Another mechanism for specificity of virulent phages could be related to inhibition of the general phage defense mechanism built up by the CRISPR-Cas system. Recent studies showed that the CRISPR-Cas system may be an actor in the bacterial resistance to virulent phages [75].

## Conclusions

*P*. *aeruginosa*, a frequent opportunistic pathogen for humans, is abundant in waste water, together with a large variety of lytic and temperate phages. The phages have a profound influence on bacterial communities, through regulation of populations by mortality, and through modification of bacterial fitness and physiology by gene transfer and selection of resistant mutants [5,76]. We describe here *P*. *aeruginosa* phage diversity inside a single environment over a two-year period in a large African city. By comparison with published phage genomes, we show that the African phages, although belonging to already known genera, form a distinct population with important internal similarities. All the collect sites were somewhat connected which could explain that very similar phages were sampled at distant locations. It is thus likely that the phages grow inside a bacterial community that colonize the waste water system, providing here a unique view of their evolution in the environment.

The advance of high throughput sequencing technologies allows investigations of microbial populations on a large scale. Whole genome sequencing was applied to all isolated phages with high accuracy, and assembly produced a single contig. We expect that such an approach will open the way to new studies of phage-bacteria coevolution, either in natural environment or following therapeutic use of bacteriophages.

## Supporting Information

S1 Fig Plaque morphology of phage Ab22 on bacterial isolate C2-10. Both clear and turbid plaques are visible.
(TIF)

S2 Fig Determination of the DTR. A) Detail of a region showing two peaks of reads and corresponding to the existence of the DTR at both ends of the genome. B) Sequencing reads showing a fixed end.
(PDF)

S3 Fig Alignment using Geneious of five genomes of PAKP1-like phages, Ab01, Ab02, Ab08, Ab10 and Ab15. (PDF)

S4 Fig Alignment using Geneious of five genomes of KPP10-like phages Ab03, Ab04, Ab06, Ab11 and Ab17. (PDF)

S5 Fig Alignment using Geneious of 3 genomes of PB1-like phages Ab27, Ab28 and Ab29. (PDF)

S6 Fig Alignment using Geneious of 3 genomes of YuA-like phages Ab18, Ab19 and Ab20. (PDF)

S1 Table Putative function of ORFs observed in annotated phage genomes. (XLS)

S2 Table Host range of 29 Abidjan phages. (XLS)
