# Supplementary figures and images for: Investigation of a Large Collection of Pseudomonas aeruginosa Bacteriophages Collected from a Single Environmental Source in Abidjan, Côte d’Ivoire
Source: PLoS One. 2015 Jun 26;10(6):e0130548. doi: 10.1371/journal.pone.0130548 (PMC4482731; doi:10.1371/journal.pone.0130548)

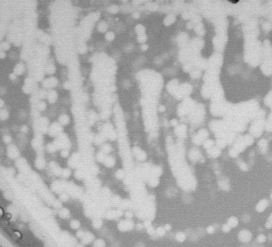

Supplement: S1 Fig — Both clear and turbid plaques are visible. (TIF) [file pone.0130548.s001.tif]

A

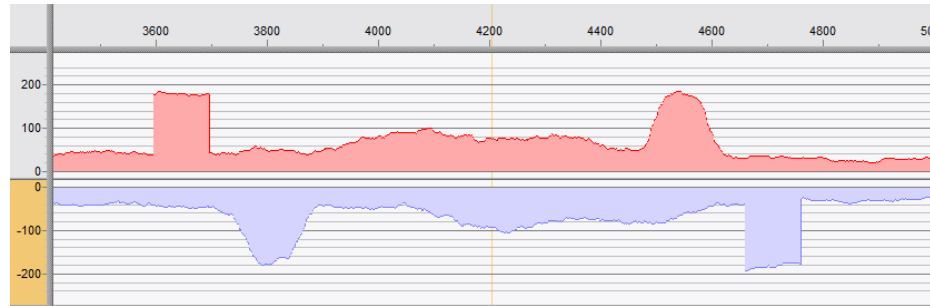

B

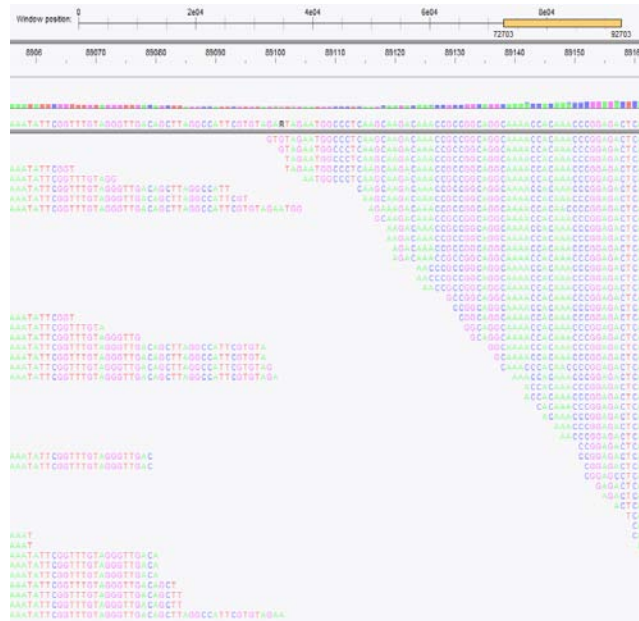

Supplement: S2 Fig — A) Detail of a region showing two peaks of reads and corresponding to the existence of the DTR at both ends of the genome. B) Sequencing reads showing a fixed end. (PDF) [file pone.0130548.s002.pdf]

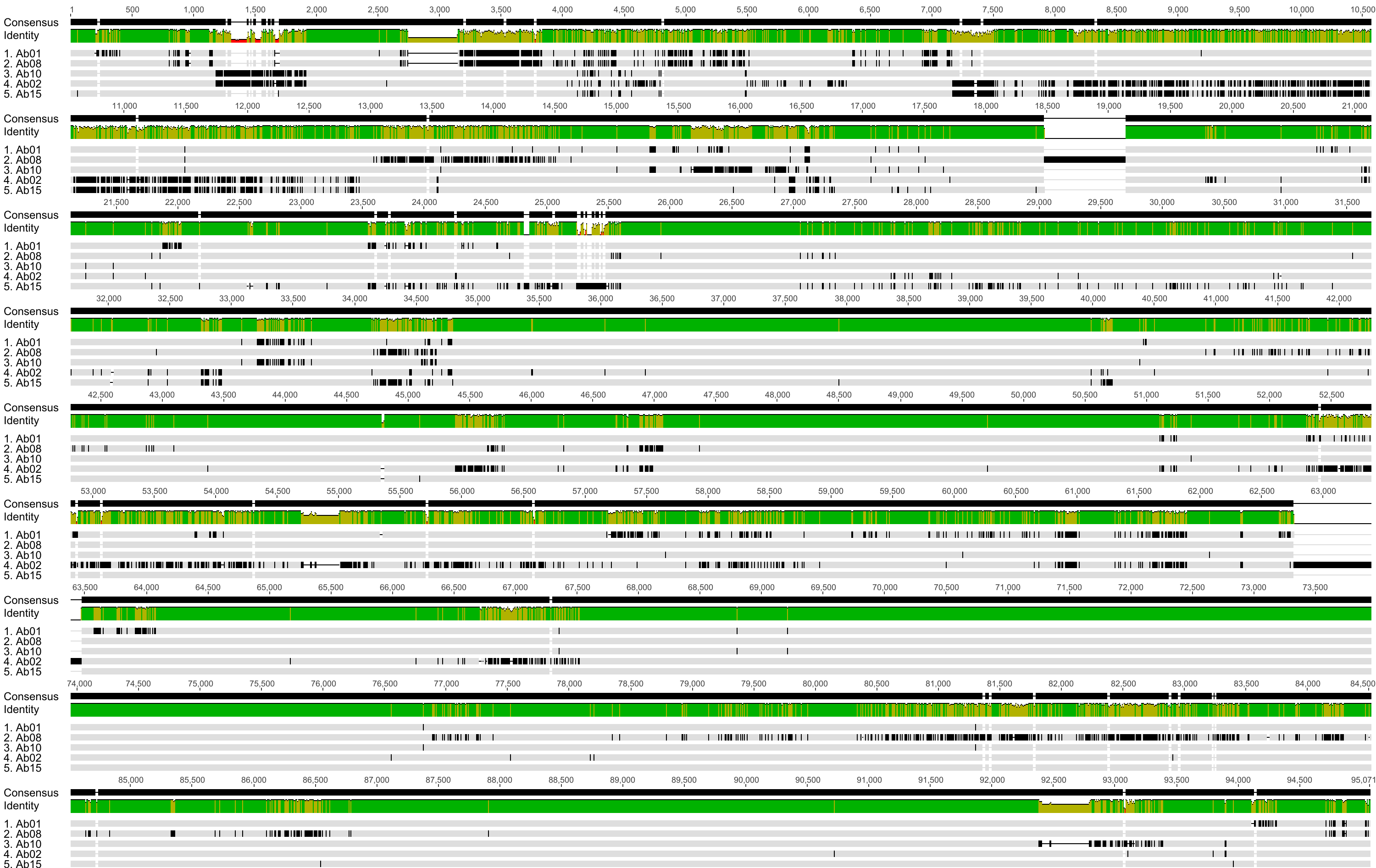

Supplement: S3 Fig — (PDF) [file pone.0130548.s003.pdf]

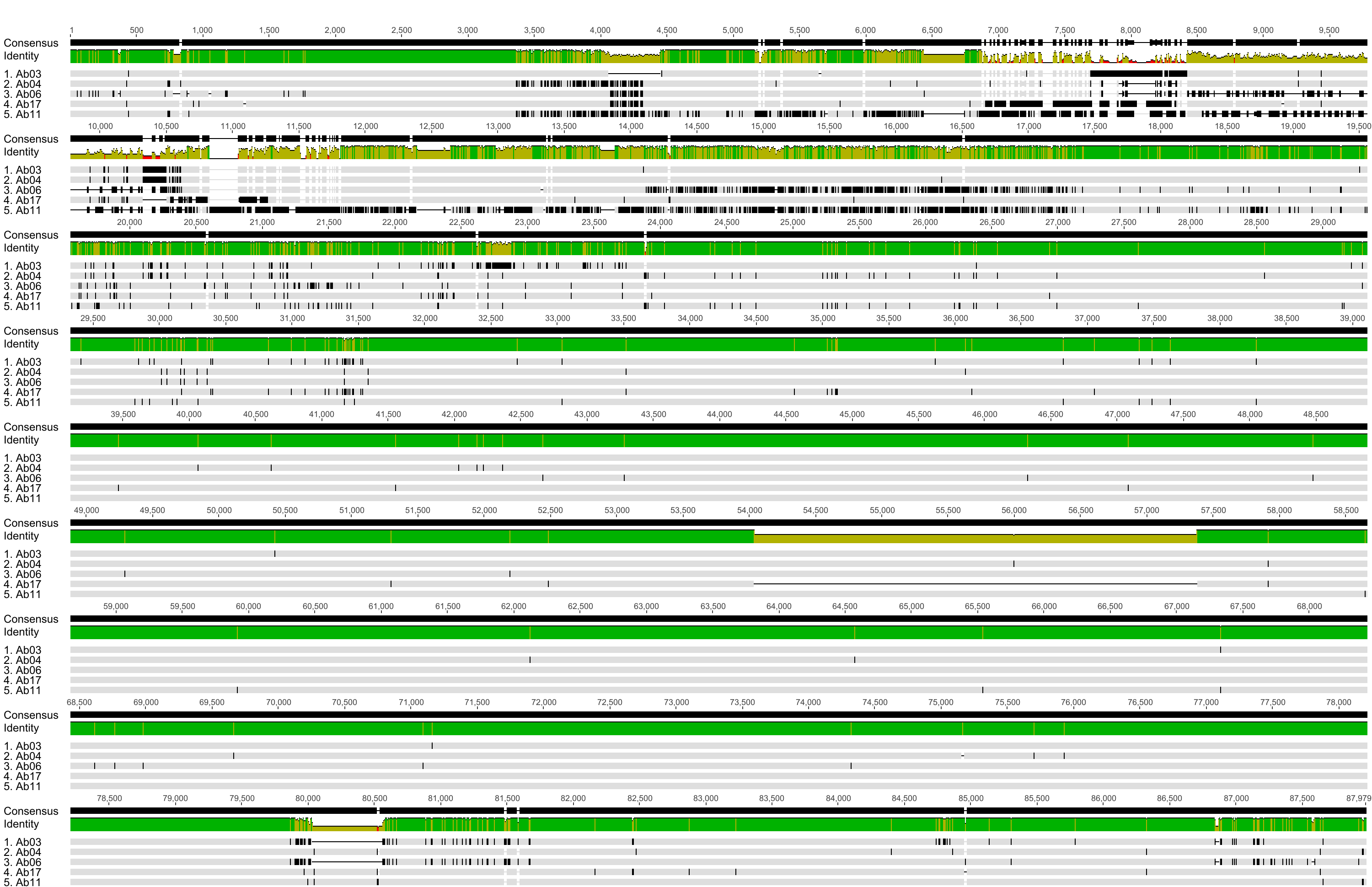

Supplement: S4 Fig — (PDF) [file pone.0130548.s004.pdf]

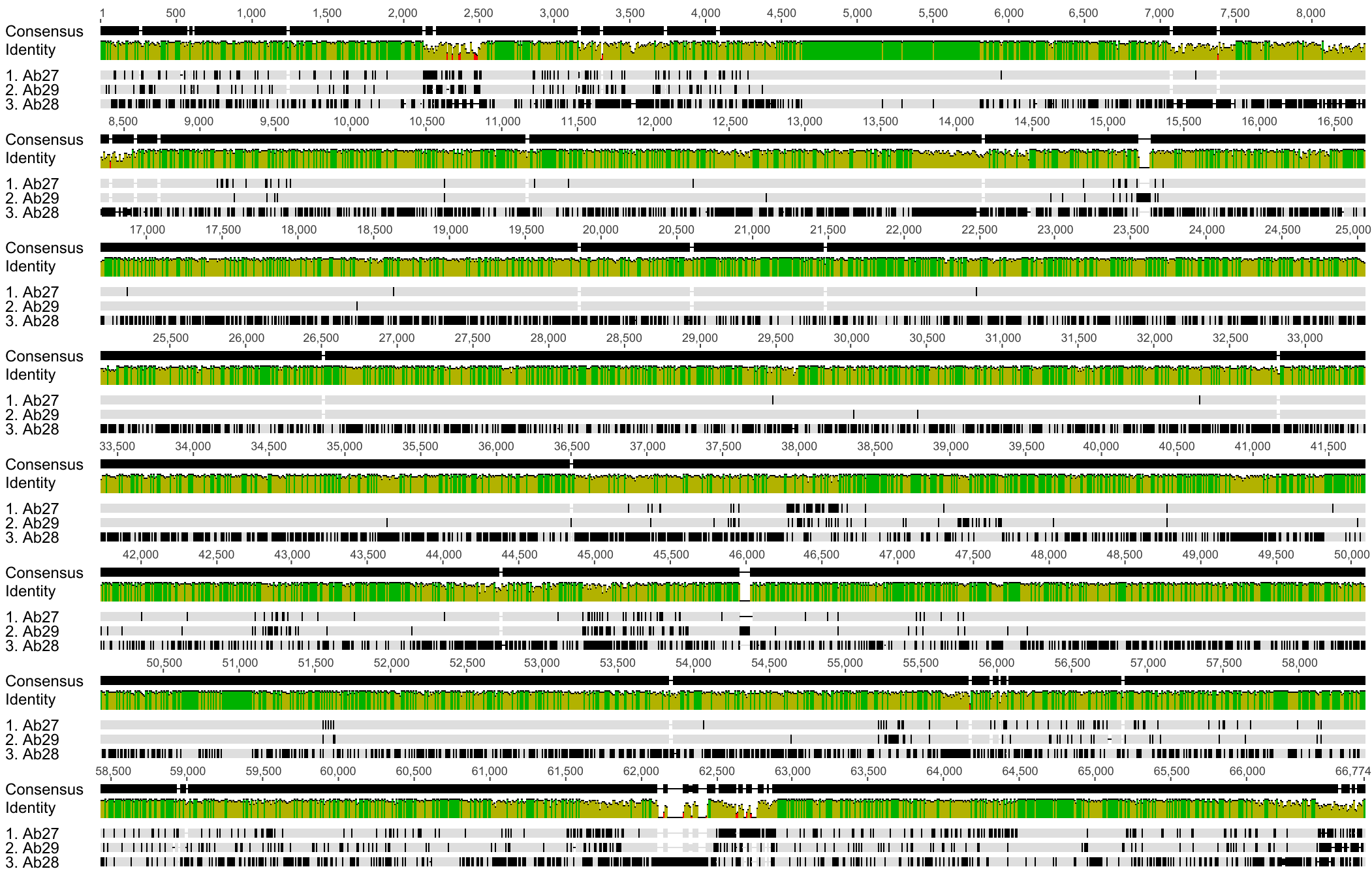

Supplement: S5 Fig — (PDF) [file pone.0130548.s005.pdf]

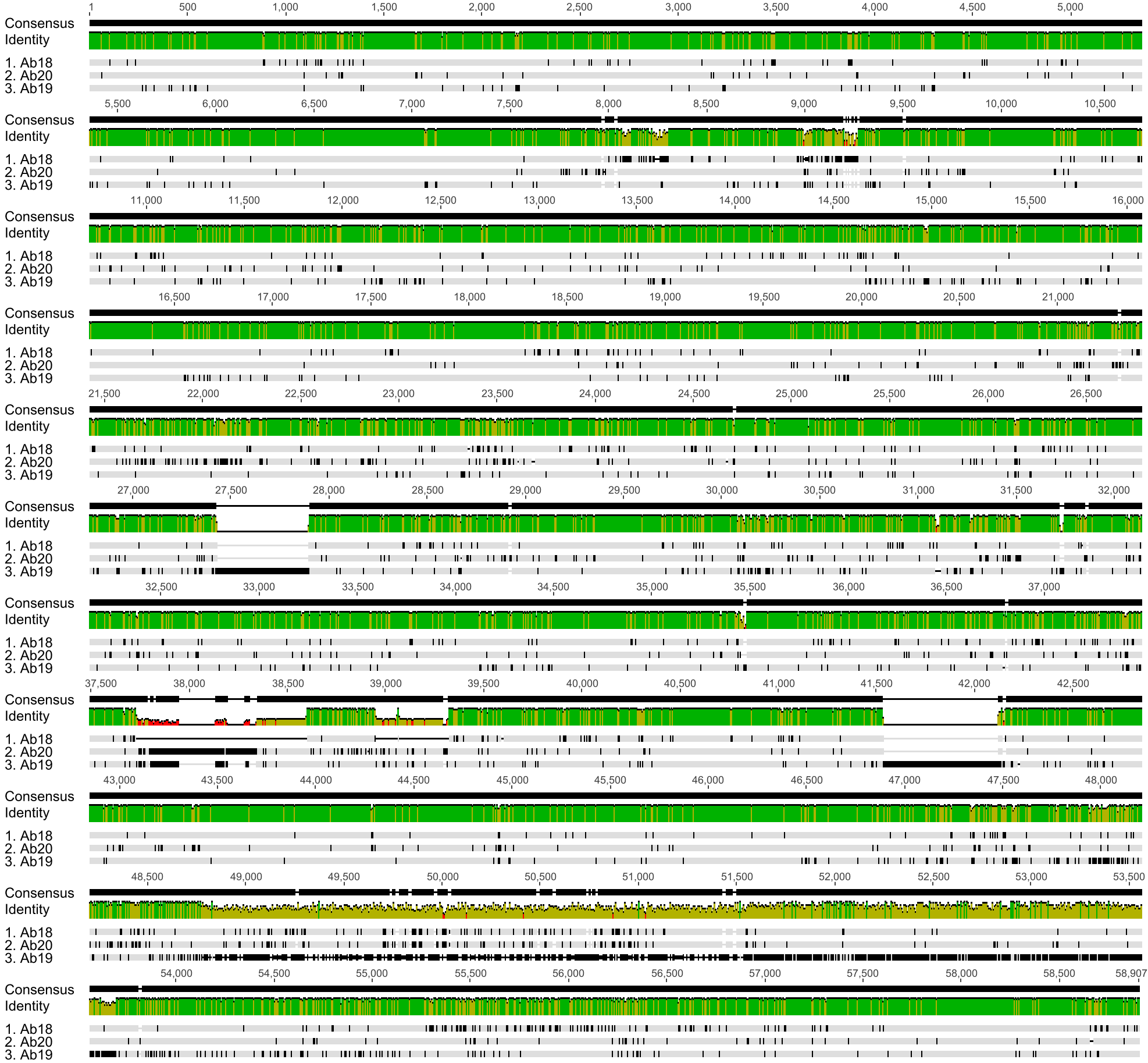

Supplement: S6 Fig — (PDF) [file pone.0130548.s006.pdf]
